# Waste to wealth: circular approaches for microbial pigment production

**DOI:** 10.3389/fmicb.2026.1815563

**Published:** 2026-07-09

**Authors:** Moitrayee Devi, Arnabjyoti Deva Sarma, Suresh Deka, Rajashree Bhattacharyya, Deep Prakash Parasar

**Affiliations:** 1 Faculty of Allied and Healthcare Sciences, Assam Down Town University, Guwahati, Assam, India; 2 Faculty of Sciences, Assam Down Town University, Guwahati, Assam, India; 3 Programme of Biotechnology, Faculty of Sciences, Assam Down Town University, Guwahati, Assam, India

**Keywords:** biomass, microbial, natural, pigments, waste

## Abstract

The increasing demand for natural and sustainable colorants has accelerated interest in microbial pigments as alternatives to synthetic dyes. Diverse microorganisms, including bacteria, fungi, yeasts, microalgae, and actinomycetes, produce pigments such as carotenoids, melanins, prodigiosin, violacein, and phycobiliproteins with applications in food, pharmaceuticals, cosmetics, textiles, and biomedicine. Despite their potential, large-scale microbial pigment production remains constrained by high costs, refined substrate dependency, and downstream processing challenges. This review summarizes recent advances in microbial pigment production using agro-industrial residues, food processing wastes, lignocellulosic biomass, and other organic waste streams within a circular bioeconomy framework. Key microbial sources, waste-derived substrates, and bioprocess strategies are discussed alongside techno-economic, environmental, and regulatory considerations. Current bottlenecks and emerging approaches, including metabolic engineering, synthetic biology, and integrated biorefinery concepts, are highlighted as future directions to enhance sustainability and industrial scalability.

## Graphical abstract

**Figure:**
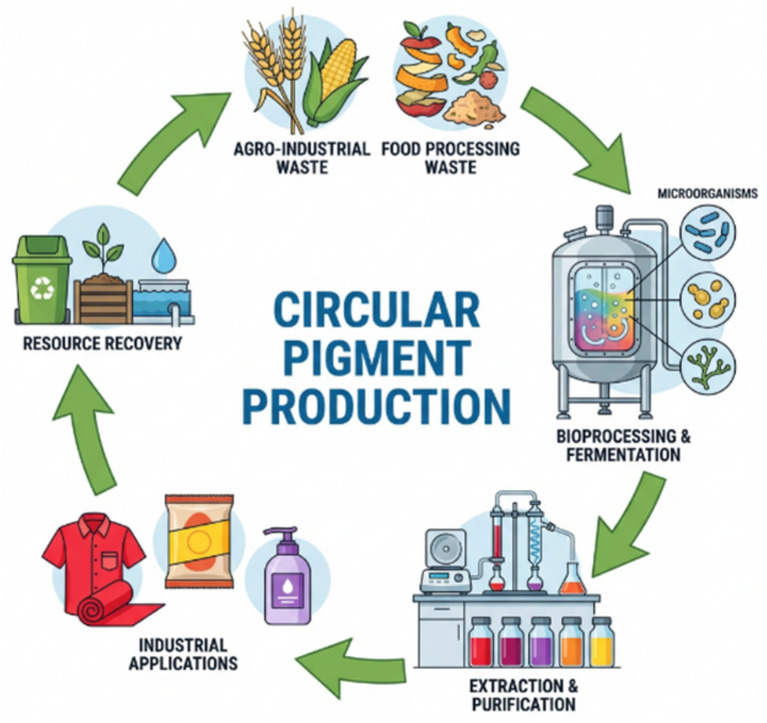

## 1 Introduction

Pigments play a critical role across multiple industrial sectors, including food, pharmaceuticals, cosmetics, textiles, packaging, and nutraceuticals. Historically, the global pigment market has been dominated by synthetic colorants due to their low production cost, high stability, and broad color range. However, growing awareness of the adverse health and environmental impacts associated with synthetic pigments, such as toxicity, carcinogenicity, and poor biodegradability has led to increasing regulatory scrutiny and consumer resistance (Agarwal et al., 2023; Devi et al., 2024). Consequently, there is a strong global shift toward natural pigments derived from plants, animals, and microorganisms.

Recent market analyses highlight the increasing demand for natural colorants. Which estimate the global natural dyes and pigments market at USD 4.83 billion in 2024 and predict growth to USD 7.95 billion by 2033, representing an annual growth rate of 5.8%. Among the various sources, microbial-derived colorants are projected to exhibit the highest growth rate, expanding at an estimated CAGR of 6.7% throughout the projected period. The market growth is being supported by rising consumer preference for sustainable and naturally sourced products, and the widening application of bio-based pigments in multiple industrial sectors, including food, pharmaceuticals, cosmetics, and textiles (Negi, 2025; Grand View Research, 2025).

The demand for natural pigments has expanded rapidly, driven by consumer preferences for clean-label products, stricter food safety regulations, and sustainability goals outlined in global frameworks such as the United Nations Sustainable Development Goals (SDGs; Devi et al., 2024; Negi, 2025). Despite their advantages, conventional natural pigment sources particularly plant- and animal-derived pigments pose significant challenges, including seasonal variability, land and water use intensity, low extraction yields, and competition with food crops (Negi, 2025; Devi et al., 2025b). These limitations have prompted the exploration of alternative, sustainable pigment sources. Microbial pigments have emerged as promising substitutes due to their structural diversity, bioactivity, scalability, and independence from climatic conditions (Devi et al., 2025b). Nevertheless, large-scale microbial pigment production is still constrained by high fermentation costs, energy-intensive downstream processing, and reliance on refined substrates, which can undermine environmental and economic sustainability (Chavan et al., 2025). Addressing these challenges is essential for the commercial viability of microbial pigments and for aligning pigment production with circular and sustainable industrial practices.

The generation of agro-industrial and waste products from food processing has increased substantially around the world, creating major environmental and disposal concerns. Worldwide, an estimated 1.3 billion tonnes of food are lost or wasted each year (Balan et al., 2023). The annual production of over 100 billion tonnes of lignocellulosic biomass is linked to agricultural-related activities (Sanchez and Cardona, 2008). Additionally, the dairy and brewing industry generate huge amounts of by-products that include whey and brewer’s spent grain, respectively, which are often underutilized due to their high level of nutrients (Sadh et al., 2018). Valorization of these residues as fermentation substrates is a sustainable approach to minimize the accumulation of waste materials and increase the economic feasibility of bacterial pigment production (Sadh et al., 2018).

**Figure 1:**
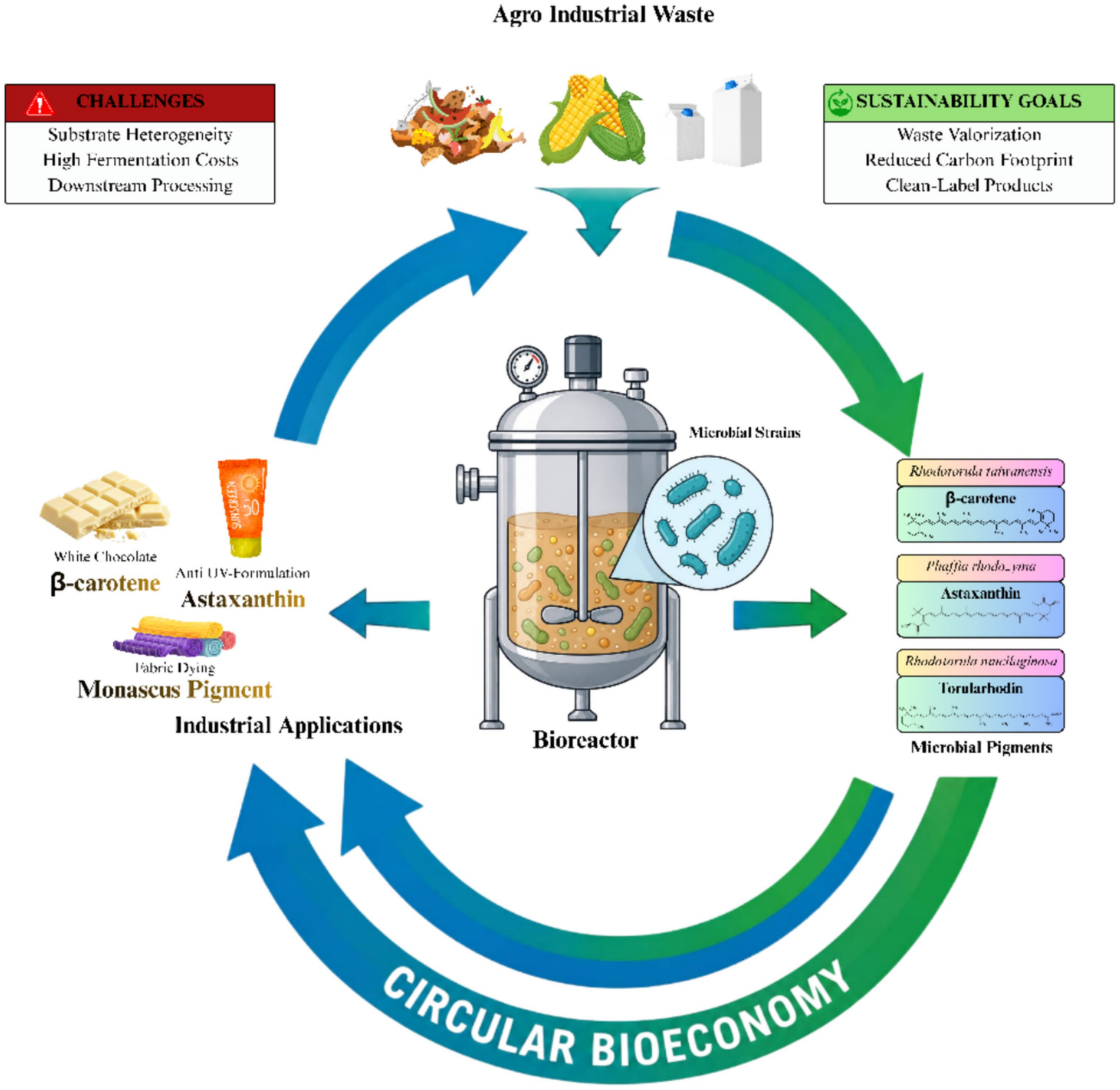
Circular bioeconomy for microbial pigment production.

The waste-to-wealth concept is rooted in the principles of circular economy, which emphasize minimizing waste generation, maximizing resource efficiency, and closing material loops through reuse, recycling, and valorization (Pal et al., 2024; Reddy et al., 2023). In the context of the bioeconomy, this approach focuses on converting organic waste streams into high-value bioproducts using biological systems and biotechnological processes (Pal et al., 2024; Anbarasu et al., 2026). Agro-industrial residues, food processing by-products, lignocellulosic biomass, dairy waste, molasses, fruit peels, and brewery effluents represent abundant, low-cost substrates rich in carbon, nitrogen, and micronutrients (Rațu et al., 2023). Improper disposal of these wastes contributes to greenhouse gas emissions, soil and water pollution, and economic losses. Microbial bioprocesses offer an environmentally benign strategy to valorize such waste streams by converting them into value-added compounds, including enzymes, organic acids, biofuels, and pigments (Lad et al., 2022). Integrating microbial pigment production into a circular bioeconomy framework enables simultaneous waste management and bioproduct generation, thereby enhancing process sustainability and economic feasibility (Możejko-Ciesielska, 2025). Pigments such as carotenoids, prodigiosin, violacein, melanin, and Monascus pigments have been successfully produced using diverse waste-derived substrates, demonstrating the potential of waste-to-wealth strategies in reducing production costs and environmental footprints (Panesar et al., 2015; Usmani et al., 2020). This alignment of industrial biotechnology with circular economy principles positions microbial pigment production as a key contributor to sustainable manufacturing and green innovation (Figure 1).

The majority of studies on waste-based microbial pigment production have investigated agro-industrial residues, food-processing by-products, and lignocellulosic biomass as substrates for microbial cultivation and pigment biosynthesis (Grewal et al., 2022; Panesar et al., 2015). These organic wastes are rich in carbon and nutrients and have been widely explored for the production of value-added pigments through microbial fermentation (Grewal et al., 2022; Panesar et al., 2015). In addition to organic wastes, wastewater streams have also been utilized for the cultivation of microalgae and the production of several value-added products, including carotenoids such as *β*-carotene, astaxanthin, and fucoxanthin (Gupta et al., 2024). However, the present review is intentionally limited to organic waste streams that can be directly utilized as microbial substrates for pigment production and waste valorization.

This review aims to provide a comprehensive and critical synthesis of recent advances in microbial pigment production within the framework of waste-to-wealth and circular bioeconomy concepts. Specifically, the review focuses on (i) major classes of microbial pigments and their industrial relevance, (ii) types of agro-industrial and food wastes utilized as fermentation substrates, (iii) microbial strains and bioprocess strategies employed for pigment synthesis, and (iv) techno-economic, environmental, and regulatory considerations influencing large-scale implementation. Furthermore, this review highlights current challenges associated with waste-based pigment production, including substrate heterogeneity, process optimization, pigment recovery, and quality standardization. Emerging trends such as metabolic engineering, synthetic biology, and integrated biorefinery approaches are also discussed as future directions to enhance productivity and sustainability. By consolidating existing knowledge and identifying research gaps, this review seeks to support the development of scalable, eco-friendly, and economically viable microbial pigment production systems that contribute to global sustainability goals and circular bioeconomy initiatives.

Several recent reviews have comprehensively discussed microbial pigment production, biosynthetic diversity, industrial applications, and associated challenges, including the review by Rather et al., which primarily emphasized microbial pigment sources, production technologies, and industrial prospects (Rather et al., 2023). However, comparatively limited attention has been given to the integration of waste-derived feedstocks and circular bioeconomy principles for sustainable pigment biomanufacturing. In contrast to earlier reviews, the present review specifically focuses on the waste-to-wealth paradigm, critically synthesizing recent evidence on the utilization of agro-industrial residues, food processing by-products, lignocellulosic biomass, marine wastes, and municipal organic wastes as alternative substrates for microbial pigment production. Furthermore, this review uniquely integrates pretreatment and valorisation strategies, fermentation approaches for heterogeneous waste streams, techno-economic analysis (TEA), life cycle assessment (LCA), process scalability, and downstream sustainability considerations within a unified framework. By consolidating these multidisciplinary aspects, the present review aims to provide a more application-oriented and sustainability-focused perspective for advancing industrial-scale microbial pigment production under circular bioeconomy frameworks.

## 2 Microbial pigments: an overview

Microbial pigments comprise a diverse group of biologically synthesized colorants produced by bacteria, fungi, yeasts, microalgae, and actinomycetes. Unlike synthetic dyes, these natural pigments are increasingly valued for their multifunctional biological activities, and eco-friendly nature, making them highly relevant in modern industrial and biomedical applications (Rather et al., 2023). Microbial pigments are a structurally and functionally diverse group of natural colorants synthesized as secondary metabolites by a wide range of microorganisms. Based on their chemical structure, biosynthetic pathway, solubility, and biological function, microbial pigments are broadly classified into carotenoids, melanin’s, prodigiosin, violacein, phycobiliproteins, and several other minor pigment groups (Devi et al., 2024). This classification is essential for understanding their ecological roles and industrial potential.

### 2.1 Carotenoids

Carotenoids are one of the most extensively studied classes of microbial pigments. They are lipid-soluble isoprenoid compounds composed of a conjugated polyene chain, which imparts their characteristic yellow, orange, and red coloration. Microbial carotenoids include *β*-carotene, astaxanthin, lycopene, torulene, and zeaxanthin, produced by bacteria, yeasts, fungi, and microalgae (Rather et al., 2023). In microorganisms, carotenoids primarily function as antioxidants and photoprotective agents, protecting cells from oxidative stress, UV radiation, and photooxidative damage. Industrially, carotenoids are highly valued due to their nutraceutical, pharmaceutical, cosmetic, and food-colorant applications, particularly astaxanthin and *β*-carotene (Rather et al., 2023; Galasso et al., 2017).

### 2.2 Melanins

Melanins are high-molecular-weight, dark-colored pigments synthesized through the oxidative polymerization of phenolic or indolic precursors, such as tyrosine or dihydroxyphenylalanine (DOPA). Microbial melanins are commonly classified as eumelanin, pheomelanin, and allomelanin, depending on their precursor molecules (Tran-Ly et al., 2020). These pigments are produced by diverse microorganisms, including bacteria (*Streptomyces*, *Bacillus*), fungi (*Cryptococcus*, *Aspergillus*), and actinomycetes. Melanin plays a crucial role in UV protection, thermal tolerance, metal chelation, and resistance to enzymatic degradation, enabling microbial survival under extreme environmental conditions. Due to their radioprotective, antioxidant, and bioelectronic properties, microbial melanins are increasingly explored for applications in biomedicine, cosmetics, and environmental remediation (Pandey et al., 2023; Tsouko et al., 2023).

### 2.3 Prodigiosin

Prodigiosin is a red tripyrrole pigment predominantly produced by *Serratia marcescens*, *Streptomyces*, and other marine and soil bacteria. Structurally, prodigiosin belongs to the prodiginine family, characterized by a linear tripyrrole ring system (Islan et al., 2022). Biologically, prodigiosin functions as a competitive and defensive metabolite, exhibiting potent antibacterial, antifungal, antiprotozoal, immunosuppressive, and anticancer activities (Islan et al., 2022). Unlike many pigments used solely as colorants, prodigiosin has attracted strong research interest due to its therapeutic potential, particularly in apoptosis induction in cancer cells (Li et al., 2018). However, large-scale commercialization of these pigments remains challenging due to stability and toxicity concerns, prompting interest in metabolic engineering and sustainable production strategies.

### 2.4 Violacein

Violacein is a purple-violet bisindole pigment synthesized from L-tryptophan by bacteria such as *Chromobacterium violaceum*, *Janthinobacterium lividum*, and *Pseudoalteromonas* species. This pigment is produced via a highly regulated biosynthetic pathway involving multiple enzymatic steps (Füller et al., 2016). Violacein exhibits a broad spectrum of biological activities, including antibacterial, antiviral, antiparasitic, antioxidant, and anticancer effects. Ecologically, violacein contributes to microbial competition and quorum-sensing-mediated defense mechanisms (Zhang et al., 2025). Due to its strong bioactivity and vivid coloration, violacein is being explored for pharmaceutical development, antimicrobial coatings, and eco-friendly textile dyes, though challenges related to solubility and yield remain (Ahmed et al., 2021).

### 2.5 Phycobiliproteins

Phycobiliproteins are water-soluble, fluorescent protein pigments found mainly in cyanobacteria and red algae. The major types include phycocyanin (blue), phycoerythrin (red), and allophycocyanin, which function as accessory light-harvesting pigments in photosynthesis (Dagnino-Leone et al., 2022). Unlike most microbial pigments, phycobiliproteins are protein–chromophore complexes, conferring exceptional fluorescence and spectral properties. These pigments are widely used as natural food colorants, fluorescent probes in biomedical diagnostics, flow cytometry, and immunoassays. Among them, phycocyanin has gained commercial success due to its antioxidant, anti-inflammatory, and neuroprotective properties (Kuddus et al., 2013; Madji et al., 2025; Pagels et al., 2022).

## 3 Microbial sources

### 3.1 Pigment production by bacteria

Bacteria represent an important and versatile source of natural pigment production. Owing to their short generation time, rapid growth, and ease of genetic manipulation, bacteria are often preferred over fungi for pigment biosynthesis (Narsing Rao et al., 2017). Bacterial pigment production systems are highly adaptable and efficient, enabling rapid pigment synthesis across a wide range of fermentation media. Compared to synthetic dye production, bacterial processes offer higher productivity, lower energy requirements, and greater scalability, making them economically attractive for industrial applications (Venil et al., 2013; Kumar et al., 2022). A wide diversity of bacterial genera are known to synthesize pigments with distinct chemical structures and bioactivities. These include *Chromobacterium*, *Janthinobacterium*, *Alteromonas*, *Collimonas*, *Duganella*, *Pseudoalteromonas*, *Iodobacter*, *Rhizobium*, *Pseudomonas*, *Modestobacter*, *Streptomyces*, *Erwinia*, *Flavobacterium*, *Brevibacterium*, *Paracoccus*, *Pantibacter*, and *Serratia* spp. Collectively, these microorganisms produce an array of pigments such as prodigiosin, violacein, melanin, carotenoids, pyocyanin, zeaxanthin, and astaxanthin, among others (Celedón and Díaz, 2021). Given their diverse chromatic properties and functional bioactivities, bacterial pigments have attracted increasing research attention for applications in food, pharmaceuticals, cosmetics, textiles, and biomedical fields, including to evaluate their production efficiency, stability, and industrial applicability.

Purple non-sulfur bacteria (PNSB) which is a group of anoxygenic phototrophic microorganisms have attracted considerable interest because of their broad metabolic capabilities. These bacteria can utilize photoheterotrophic, photoautotrophic, chemoautotrophic or chemoheterotrophic modes of growth, depending on environmental conditions (Dhar et al., 2023; Assega et al., 2025). This bacterium produces some photosynthetic pigments, primarily carotenoids and bacteriochlorophylls under illuminated anaerobic or microaerobic conditions, which are responsible for light capture and energy conversion (Dhar et al., 2023).

PNSB has a capacity to use a wide range of organic compounds, including organic acids, carbohydrates, alcohols, fatty acids, and amino acids, during photoheterotrophic growth (Dhar et al., 2023; Assega et al., 2025). The broad physiological versatility of these microorganisms has encouraged their application in wastewater treatment, for simultaneous contaminant removal and biomass production (Dhar et al., 2023; Assega et al., 2025). The biomass generated by PNSB contains can be utilized as a source of valuable bioproducts, including carotenoids, single-cell protein, coenzyme Q10, and polyhydroxyalkanoates (Lu et al., 2021; Assega et al., 2025). Furthermore, the capacity of these bacteria to convert organic compounds and nutrients from wastewater into useful bioproducts complements current efforts toward resource recovery and sustainable circular bioeconomy systems (Lu et al., 2021; Assega et al., 2025). Therefore, PNSB are recognized as an important microbial source of natural pigment production and considered together with other microbial sources of natural pigments (Dhar et al., 2023; Assega et al., 2025).

### 3.2 Pigment production by fungi and yeasts

Fungi and yeasts constitute a major group of pigment-producing microorganisms, offering a wide range of natural colorants with diverse chemical structures and functional properties. Among filamentous fungi, several taxonomic families have been reported as prolific pigment producers, including *Sordariaceae, Monascaceae, Trichocomaceae, Nectriaceae, Hypocreaceae, Pleosporaceae, Cordycipitaceae, Xylariaceae, Chaetomiaceae, and Chlorociboriaceae,* as well as notable species such as *Penicillium oxalicum* and *Blakeslea trispora* (Lagashetti et al., 2019). In addition, various yeast genera, including *Rhodotorula, Xanthophyllomyces, Pichia, Sporobolomyces, and Sporidiobolus*, are well recognized for their ability to synthesize valuable pigments. These pigment-producing fungi and yeasts generate a broad spectrum of colored compounds, such as monascin, carotenoids, melanins, flavins, anthraquinones, violacein, phenazines, and quinones, contributing to yellow, orange, red, brown, and black hues. Several of these pigments exhibit bioactive properties, including antioxidant, antimicrobial, and anticancer activities, which enhance their industrial relevance (Lagashetti et al., 2019). Furthermore, studies have shown that certain marine-derived and endophytic fungi, including genera such as *Trimmatostroma*, *Phaeotheca*, *Hortaea*, and *Halorosellinia*, are also capable of pigment production, highlighting the ecological diversity and untapped potential of fungal pigment sources (El-Bondkly et al., 2021). Many pigments derived from these fungal and yeast strains have already found applications in food, pharmaceutical, cosmetic, textile, and biotechnological industries, driven by their natural origin, stability, and functional properties.

### 3.3 Pigments from microalgae

Microalgae represent a prolific and sustainable source of natural pigments with significant industrial and biomedical relevance. Several microalgal genera, including *Nostoc, Dunaliella, Scenedesmus, Nannochloropsis, Haematococcus, Muriellopsis, Chlorella, Phaeodactylum, Spirulina (Arthrospira), Porphyridium, Agardhiella,* and *Polysiphonia*, are known to produce diverse groups of pigments such as carotenoids, chlorophylls, and phycobiliproteins (PBPs) (Pailliè-Jiménez et al., 2020). Phycobiliproteins are non-toxic, water-soluble protein pigments predominantly found in Rhodophyta (red algae), Cyanobacteria, and Cryptophyta, and are particularly valued for their intense coloration and fluorescence properties. Due to their strong light absorbance, high fluorescence efficiency, and potent antioxidant and free radical scavenging activities, phycobiliproteins have been extensively utilized in the food, cosmetic, pharmaceutical, and biomedical industries (García-Gómez et al., 2025). In addition to PBPs, microalgae produce a range of antioxidant pigments, which contribute to their broad spectrum of bioactivities. These pigments exhibit anti-inflammatory, antiangiogenic, neuroprotective, hepatoprotective, antiviral, anti-obesity, antidiabetic, anticancer, and anti-osteoporotic properties (Singh et al., 2025). Furthermore, microalgal pigments have been reported to support cardiovascular health, enhance cognitive function, protect against UV radiation, modulate immune responses, delay aging processes, and reduce the risk of certain hematological disorders (Pailliè-Jiménez et al., 2020; Ambati et al., 2019; Saini et al., 2018). Owing to these multifunctional properties, microalgal pigments possess high commercial value as natural colorants and bioactive ingredients in the nutraceutical, cosmetic, and pharmaceutical sectors. They are also widely applied in clinical research, molecular biology (e.g., fluorescent labeling), textile dyeing, painting and color industries, as well as in the food industry as colorants and in animal feed formulations, particularly for poultry (Aizpuru and González-Sánchez, 2024). Despite their immense potential, large-scale pigment production from microalgae remains challenged by factors such as cultivation costs, productivity limitations, and downstream processing inefficiencies. Addressing these bottlenecks through genetic engineering, metabolic pathway optimization, and advanced cultivation strategies is critical for achieving economically viable and sustainable pigment production (Pailliè-Jiménez et al., 2020; Saini et al., 2020).

### 3.4 Pigments produced from actinomycetes

Actinomycetes produce a wide spectrum of pigments exhibiting diverse colors and significant industrial relevance. Among the most well-documented actinomycete-derived pigments are melanin (black to brown), carotenoids (yellow to orange), prodigiosins (red), and actinorhodin(blue). These pigments are predominantly synthesized by species of the genus *Streptomyces*, including *Streptomyces glaucescens*, *Streptomyces albidoflavus*, and *Streptomyces coelicolor* (Díez et al., 2025; Melzoch et al., 1997; Liu et al., 2017). Melanin is a dark, high-molecular-weight pigment produced by several *Streptomyces* species and is highly valued for its exceptional photoprotective and antioxidant properties. Its remarkable stability and resistance to environmental degradation enhance its suitability for industrial applications (Díez et al., 2025). In the cosmetic industry, melanin is widely explored for incorporation into sunscreen formulations and anti-aging products. Additionally, melanin serves as a natural black or brown dye in the textile industry and has promising biomedical applications, including wound dressings and photoprotective therapeutic materials (El-Naggar and El-Ewasy, 2017). Carotenoids synthesized by *Streptomyces* species, such as *S. albidoflavus*, include compounds like *β*-carotene, lycopene, and astaxanthin, which impart yellow, orange, and red hues. These pigments are well recognized for their strong antioxidant activity and are extensively utilized in the food industry as natural colorants and nutritional additives, offering a non-toxic alternative to synthetic dyes (Díez et al., 2025). In cosmetics, carotenoids are incorporated into skincare formulations to mitigate oxidative damage and improve skin health. Furthermore, their role in reducing oxidative stress has generated interest in pharmaceutical applications, particularly for the prevention and management of diseases such as cancer and cardiovascular disorders (Sandmann, 2021). Prodigiosins are red tripyrrole pigments produced by actinomycetes such as *Streptomyces coelicolor* and are distinguished by their potent antimicrobial and anticancer activities. These bioactive compounds have gained considerable attention in pharmaceutical research as promising candidates for antimicrobial and anticancer drug development. Beyond therapeutics, prodigiosin’s are also explored in cosmetics as natural red colorants with added biofunctional benefits. Their antimicrobial properties further enhance their value in the textile industry, where they can be used to produce antibacterial fabrics (Liu et al., 2017). Actinorhodin, a distinctive blue pigment also produced by *Streptomyces coelicolor*, possesses both esthetic appeal and functional bioactivity, particularly antimicrobial properties. This pigment shows strong potential as a natural blue dye for textile applications, offering a biodegradable and environmentally safer alternative to synthetic blue dyes. In the pharmaceutical sector, actinorhodin’s antimicrobial activity supports its investigation as a candidate for the development of novel antibacterial agents (Díez et al., 2025).

### 3.5 Emerging and underexplored microbial platforms

In addition to well-known pigment-producing microorganisms, other microbial platforms may provide new pathways for pigment bioprocessing. Phototrophic microorganisms produce pigments like carotenoids and bacteriochlorophylls, which help with photosynthesis and photoprotection. Extremophilic microbes, such as halophiles and thermophiles, have been identified as possible sources of pigments and important biomolecules due to their ability to adapt to extreme environments (Możejko-Ciesielska, 2025). The investigation of these less common microbial resources may aid in the development of novel pigments and expand their biotechnological uses (Norena-Caro et al., 2025; Możejko-Ciesielska, 2025).

## 4 Biological functions and industrial relevance of microbial pigments

Microbial pigments are not merely color-imparting compounds but serve as multifunctional secondary metabolites that play crucial roles in microbial survival, ecological interactions, and adaptation to environmental stress. Their diverse biological functions underpin their expanding industrial relevance, positioning microbial pigments as sustainable alternatives to synthetic dyes across multiple sectors.

### 4.1 Biological functions of microbial pigments

#### 4.1.1 Photoprotection and UV shielding

Microbial pigments play a significant role in photoprotection and UV shielding due to their inherent ability to absorb ultraviolet radiation and counteract UV-induced oxidative stress. Pigments such as melanin, carotenoids, scytonemin, violacein, and prodigiosin act as natural photoprotective agents by dissipating harmful UV energy and scavenging reactive oxygen species, thereby preventing DNA damage, protein oxidation, and membrane destabilization (Kiki, 2023). Microbial melanins exhibit broad-spectrum UV absorption and strong antioxidant properties, making them particularly effective as biological UV filters (Brenner and Hearing, 2008). Cyanobacterial pigments such as scytonemin function as extracellular UV screens, enabling survival under prolonged solar radiation in extreme environments (Sen and Mallick, 2022). Additionally, carotenoids produced by bacteria and fungi contribute to photoprotection by quenching singlet oxygen and reducing photooxidative damage (Gammone et al., 2015). Owing to their natural origin, biodegradability, and low toxicity, microbial pigments are increasingly explored as sustainable alternatives to synthetic UV filters in cosmetic, pharmaceutical, and textile applications.

#### 4.1.2 Antioxidant and oxidative stress mitigation

Microbial pigments exhibit strong antioxidant potential and play a crucial role in mitigating oxidative stress by neutralizing reactive oxygen species (ROS) generated during metabolic processes and environmental stress conditions. Pigments such as carotenoids, melanins, prodigiosin, violacein, and phenazine derivatives function as effective free radical scavengers, lipid peroxidation inhibitors, and redox stabilizers, thereby protecting cellular components including DNA, proteins, and membranes from oxidative damage (Chavan et al., 2025). Microbial melanins demonstrate exceptional redox-buffering capacity and metal-chelating properties, enabling them to (Sandmann, 2021). Similarly, carotenoids produced by bacteria and fungi contribute to oxidative stress mitigation by quenching singlet oxygen and reducing ROS accumulation, enhancing cellular resilience and survival (Martínez-Cámara et al., 2021). Due to their natural origin, biodegradability, and low cytotoxicity, microbial pigments are increasingly explored as sustainable antioxidant agents in pharmaceutical, nutraceutical, cosmetic, and food preservation applications (Venil et al., 2020b).

#### 4.1.3 Ecological competition and antimicrobial defense

Microbial pigments play a crucial role in ecological competition and antimicrobial defense by providing producing organisms with a selective advantage in diverse and often resource-limited environments. Many pigmented secondary metabolites, including prodigiosin, violacein, phenazines, pyocyanin, and melanins, exhibit broad-spectrum antimicrobial activity against competing bacteria, fungi, and protozoa, thereby suppressing rival populations and facilitating niche establishment (Narsing Rao et al., 2017). These pigments interfere with essential cellular processes such as membrane integrity, electron transport, and quorum sensing, contributing to competitive dominance within complex microbial communities (Soliev et al., 2011; Mavrodi et al., 2006). In addition to direct antimicrobial effects, certain pigments function as redox-active compounds that generate or neutralize reactive oxygen species, further inhibiting competitor survival under environmental stress conditions (Cordero et al., 2017; Price-Whelan et al., 2007). Pigment production is often tightly regulated in response to environmental cues, including nutrient limitation and population density, underscoring their role as adaptive traits in microbial ecology (Pierson III and Pierson, 2010; Kumar et al., 2022). Collectively, these properties highlight microbial pigments as multifunctional molecules central to ecological competition, community structuring, and natural antimicrobial defense strategies.

#### 4.1.4 Photosynthesis and energy harvesting

In photosynthetic microorganisms, pigments play a central role in light harvesting and energy transfer by capturing photons and funneling excitation energy to reaction centers. Chlorophylls absorb primarily in the blue and red regions of the spectrum, while accessory pigments such as phycobiliproteins significantly broaden the usable light range, enabling efficient photosynthesis under fluctuating and low-light conditions (Orlandi et al., 2022). Phycobiliproteins including phycocyanin and phycoerythrin are organized into phycobilisomes in cyanobacteria and red algae, where they function as highly efficient antenna complexes that transfer excitation energy to photosystem II and photosystem I with minimal energy loss (Stadnichuk and Kusnetsov, 2023). This pigment diversity enhances photosynthetic plasticity and allows microorganisms to occupy ecological niches with distinct spectral light environments, contributing to their evolutionary success and primary productivity in aquatic ecosystems.

### 4.2 Industrial relevance of microbial pigment

#### 4.2.1 Food and nutraceutical industries

Microbial pigments have gained substantial industrial relevance in the food and nutraceutical sectors due to their coloring properties and bioactive effects, largely driven by increasing consumer preference for clean-label, health-promoting, and environmentally sustainable products. Carotenoids such as *β*-carotene, astaxanthin, and zeaxanthin produced by bacteria, yeasts, and microalgae are widely incorporated into foods and dietary supplements due to their proven safety, high bioavailability, and functional stability during processing and storage (Devi et al., 2024). These pigments not only impart attractive coloration but also provide significant health benefits, including antioxidant, anti-inflammatory, and immune-modulating activities, which contribute to the prevention of oxidative stress–related disorders (Devi et al., 2024). Similarly, phycocyanin derived primarily from cyanobacteria (*Arthrospira* spp.) is approved as a natural blue colorant and nutraceutical ingredient, valued for its antioxidative, anti-inflammatory, and hepatoprotective properties. The dual functionality of microbial pigments as colorants and bioactive compounds enhances their commercial appeal and aligns with regulatory and market trends favoring multifunctional, natural food additives (Dufosse et al., 2014). Collectively, these attributes position microbial pigments as key components in the development of next-generation functional foods and nutraceutical formulations.

#### 4.2.2 Pharmaceutical and biomedical applications

Microbial pigments possess a wide spectrum of bioactivities that have attracted considerable interest for pharmaceutical and biomedical applications. Pigments such as prodigiosin and violacein exhibit potent anticancer, immunomodulatory, and broad-spectrum antimicrobial activities, mediated through mechanisms including apoptosis induction, immune signaling modulation, and disruption of microbial cellular processes. These properties make them promising leads for drug discovery and therapeutic development (Tekawade et al., 2025; Escamilla-Medrano et al., 2025). In parallel, pigments such as melanin and phycobiliproteins are increasingly explored for biomedical uses including wound healing, photoprotection, antioxidant therapy, and tissue engineering. Fluorescent phycobiliproteins, notably phycocyanin and phycoerythrin, are widely employed as highly sensitive probes in flow cytometry, immunoassays, and molecular imaging due to their strong fluorescence, water solubility, and biocompatibility (Pagels et al., 2019). Collectively, these multifunctional properties position microbial pigments as valuable bioactive molecules in modern biomedical research and clinical diagnostics.

#### 4.2.3 Cosmetic and personal care products

In the cosmetic and personal care industry, microbial pigments are increasingly incorporated not only as natural colorants but also as functional ingredients offering skin-protective benefits. Carotenoids and melanins are commonly utilized in sunscreens, anti-aging formulations, and skincare products owing to their UV-absorbing capacity, antioxidant activity, and ability to mitigate oxidative stress-induced skin damage (Stanescu et al., 2025). These pigments contribute to photoprotection, prevention of premature aging, and maintenance of skin health. Importantly, the biodegradability, low toxicity, and sustainable production of microbial pigments align with the growing regulatory and consumer demand for eco-friendly and naturally derived cosmetic ingredients (Devi et al., 2024). As a result, microbial pigments are increasingly incorporated into next-generation cosmetic formulations due to their antioxidant and photoprotective properties.

#### 4.2.4 Textile, leather, and material industries

Microbial pigments have shown strong dyeing efficiency in textile and leather industries and also offer functional benefits such as antimicrobial effects and UV shielding, which are often associated with toxic effluents and highwater consumption. Pigments such as violacein, actinorhodin, prodigiosin and carotenoids have demonstrated effective dyeing performance on natural and synthetic fabrics (Devi et al., 2025b). In addition to coloration, these pigments often impart functional properties such as antimicrobial activity and UV protection, thereby enhancing the durability, hygiene, and value of textile products (Venil et al., 2020b). The integration of microbial pigments into textile and material processing supports cleaner production technologies and contributes to the development of sustainable industrial practices.

#### 4.2.5 Environmental and industrial biotechnology

Beyond their traditional role as natural colorants, microbial pigments have emerged as functionally important molecules in environmental and industrial biotechnology owing to their redox activity, metal-binding capacity, and electrochemical properties. Pigments such as melanins possess abundant functional groups (e.g., carboxyl, hydroxyl, and phenolic moieties) that enable efficient chelation of heavy metals, thereby reducing metal toxicity and facilitating microbial survival in contaminated environments (Thaira et al., 2019). This property has been widely explored in bioremediation strategies targeting metal-polluted soils and aquatic systems, where melanin-producing microbes contribute to metal immobilization and detoxification (Gadd, 2010). Similarly, phenazine pigments function as redox-active secondary metabolites that participate in extracellular electron transfer, influencing metal reduction processes and biogeochemical cycling of iron and other transition metals (Hernandez et al., 2004). In addition to environmental remediation, the electrochemical characteristics of melanins and phenazines have enabled their application in microbial fuel cells, bioelectronic interfaces, and redox-based biosensors. These pigments act as electron shuttles, enhancing electron transfer between microbial cells and electrodes, thereby improving current generation and signal transduction efficiency (Rabaey et al., 2005). Collectively, these multifunctional properties position microbial pigments at the intersection of microbiology, materials science, and environmental engineering, highlighting their growing potential in sustainable industrial technologies and next-generation bioelectronic systems (Table 1).

**Table 1:** Major microbial pigments, producing organisms, and applications.

Pigment	Colour	Major producing microorganisms	Chemical class	Key applications
Carotenoids (β-carotene, astaxanthin, lycopene)	Yellow, orange, red	*Dunaliella salina*, *Blakeslea trispora*, *Rhodotorula* spp., *Micrococcus* spp.	Terpenoids	Food colorants, nutraceuticals, antioxidants, cosmetics, feed additives (aquaculture)
Prodigiosin	Red	*Serratia marcescens*, *Streptomyces* spp.	Tripyrrole	Anticancer, antimicrobial, immunosuppressive, textile dye
Violacein	Purple	*Chromobacterium violaceum*, *Janthinobacterium lividum*	Indole-derived	Antimicrobial, anticancer, antiviral, pharmaceutical applications
Melanin	Brown, black	*Streptomyces* spp., *Bacillus* spp., *Cryptococcus neoformans*	Phenolic polymer	UV protection, radioprotection, cosmetics, bioremediation, electronics
Pyocyanin	Blue-green	*Pseudomonas aeruginosa*	Phenazine	Biosensors, antimicrobial agents, redox mediators, bioelectronics
Phycocyanin	Blue	*Spirulina platensis*, *Synechococcus* spp.	Phycobiliprotein	Natural food colorant, nutraceuticals, fluorescent markers, pharmaceuticals
Phycoerythrin	Red	*Porphyridium* spp., *Rhodomonas* spp.	Phycobiliprotein	Food colorants, cosmetics, fluorescent probes, biomedical research
Riboflavin	Yellow	*Ashbya gossypii*, *Bacillus subtilis*	Flavin	Food fortification, pharmaceutical supplements, feed additives
Monascus pigments	Yellow, orange, red	*Monascus purpureus*, *Monascus ruber*	Polyketides	Food colorants, nutraceuticals, traditional fermented foods
Actinorhodin	Blue	*Streptomyces coelicolor*	Polyketide	Antibiotic, pharmaceutical research
Flexirubin	Yellow-orange	*Flavobacterium* spp., *Cytophaga* spp.	Aryl polyene	Industrial colorants, antimicrobial potential
Scytonemin	Yellow-brown	*Nostoc* spp., *Scytonema* spp.	Indolic-phenolic	UV protection, sunscreen formulations, cosmetic applications

## 5 Conventional substrates vs. waste-derived feedstocks

### 5.1 Limitations of refined substrates in pigment production

**Figure 2:**
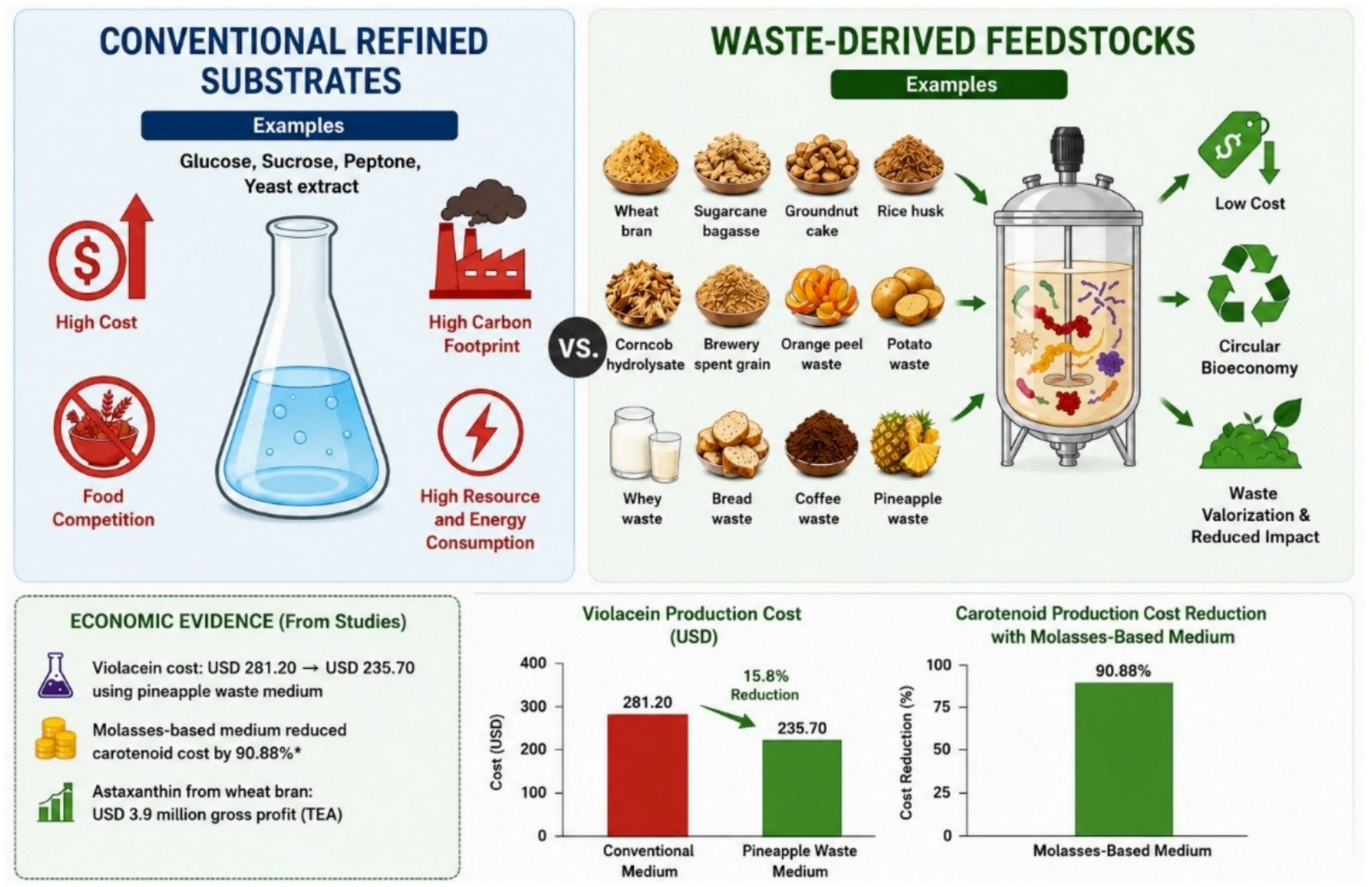
Comparison of conventional substrates vs. waste-derived feedstocks for pigment production.

Conventional microbial pigment production has traditionally relied on refined substrates such as glucose, sucrose, yeast extract, and peptone to ensure consistent microbial growth and pigment biosynthesis. Although these substrates provide controlled nutritional profiles and reproducible fermentation outcomes, their large-scale application presents several economic and environmental constraints. One of the most significant limitations is the high cost of refined media components, which may account for up to 70% of the total fermentation cost, thereby reducing the commercial feasibility of microbial pigments compared to synthetic colorants (Joshi et al., 2025). In addition to economic concerns, refined substrates often originate from food-grade agricultural resources, creating competition with human and animal nutrition systems. This competition contradicts sustainability goals and raises ethical concerns regarding resource allocation in biotechnological industries (Mishra et al., 2018). Furthermore, the production and purification of refined substrates are energy-intensive processes associated with substantial greenhouse gas emissions and water consumption, contributing to the overall environmental footprint of pigment bioprocesses (Priyanka and Madan, 2025). Another drawback is the limited adaptability of refined substrates to circular bioeconomy frameworks, as they do not support waste valorization or by-product recovery. Consequently, there is increasing interest in alternative, low-cost feedstocks that can maintain pigment yields while improving environmental sustainability and economic viability (Figure 2).

### 5.2 Types of agro-industrial and food wastes

Agro-industrial and food wastes represent abundant, renewable, and nutrient-rich feedstocks suitable for microbial pigment production. These waste materials often contain fermentable carbohydrates, proteins, lipids, minerals, and bioactive compounds that support microbial growth and secondary metabolite synthesis (Panda et al., 2026; Silva et al., 2025). Commonly explored waste feedstocks include agricultural residues such as rice bran, wheat bran, sugarcane bagasse, corn stover, fruit pomace, and vegetable peels. These lignocellulosic materials are particularly suitable for solid-state and submerged fermentation following minimal pretreatment (Blasi et al., 2023). Food processing byproducts, including dairy whey, molasses, brewery spent grains, and oilseed cakes, have also been successfully employed as substrates for pigment-producing bacteria, yeasts, and filamentous fungi (Ramesh et al., 2022). Several studies have demonstrated the efficient production of carotenoids, prodigiosin, melanin, and anthraquinone pigments using waste-derived substrates, often achieving pigment yields comparable to or exceeding those obtained with refined media (Panesar et al., 2015). The biochemical diversity of waste streams can further stimulate secondary metabolism, enhancing pigment synthesis through stress-induced or nutrient-limited conditions (Figure 2).

In microbial pigment fermentation, waste-derived substrates can fulfill different nutritional functions. For instance, molasses is commonly used as a carbon source for microbial growth due to its rich sugar content, whereas corn steep liquor is typically used as a nitrogen supplement as it contains other growth-promoting nutrients, including vitamins, amino acids, and minerals (Panesar et al., 2015). In addition, agro-industrial by-products may provide essential nutrients that facilitate microbial proliferation and pigment production (Ramesh et al., 2022).

### 5.3 Environmental and economic advantages of waste utilization

The use of agro-industrial and food wastes as fermentation feedstocks offers substantial environmental and economic advantages, positioning waste valorization as a key strategy for sustainable microbial pigment production. Economically, waste substrates significantly reduce raw material costs, as many agro-industrial residues are either low-value or disposal liabilities for processing industries. Their utilization lowers medium preparation costs and improves the overall cost-effectiveness of pigment bioprocesses (Venil et al., 2020b). From an environmental perspective, waste-based pigment production contributes to organic waste reduction, diverting large volumes of biodegradable residues from landfills and uncontrolled disposal. This approach mitigates methane emissions, reduces chemical oxygen demand (COD) loads, and minimizes soil and water pollution (Lee et al., 2024). Moreover, integrating waste streams into microbial bioprocesses aligns with circular bioeconomy principles by converting low-value residues into high-value bio-based pigments. Additionally, waste valorization promotes resource efficiency and reduces dependence on virgin agricultural inputs, thereby lowering the ecological footprint of pigment production. When combined with optimized fermentation strategies and downstream processing, waste-derived feedstocks enable the development of scalable, eco-friendly, and economically viable alternatives to synthetic pigments (Ufitikirezi et al., 2024; Figure 2).

## 6 Waste streams explored for microbial pigment production

In response to the limitations of conventional substrates various waste streams have been explored as alternative feedstock for the production of microbial pigments.

To facilitate cross-study comparison and highlight current progress in sustainable pigment biomanufacturing, a comparative summary of representative studies on waste-based microbial pigment production is provided (Table 2), including substrate type, pretreatment strategies, microbial strains, fermentation approaches, pigment productivity, recovery methods, scale of operation, applications, and associated limitations.

**Table 2:** Comparative summary of recent studies on waste-based microbial pigment production.

Waste substrate	Pretreatment method	Microorganism	Pigment produced	Fermentation mode	Pigment yield/productivity	Extraction/recovery method	Scale of operation	Application	Key limitations	Reference
Sugarcane molasses	Filtration and dilution	*Rhodotorula glutinis*	Carotenoids	Submerged fermentation (SmF)	~18–22 mg/L carotenoids	Solvent extraction (acetone/ethanol)	Laboratory	Food colorant, nutraceuticals	Variable sugar composition; contamination risk	Mata-Gómez et al., 2014
Dairy whey	Heat sterilization, lactose adjustment	*Monascus purpureus*	Monascus red pigments	SmF	Enhanced pigment production (~20–30 AU/mL)	Ethanol extraction	Laboratory	Food additives, natural dyes	Batch variability; downstream purification complexity	Vendruscolo et al., 2016
Rice bran	Milling, moisture optimization	*Monascus purpureus*	Monascus pigments	Solid-state fermentation (SSF)	High pigment intensity (yield varies with substrate composition)	Solvent extraction	Pilot/Lab scale	Food colorants, nutraceuticals	Moisture control; scale-up difficulty	Babitha et al., 2007
Corn steep liquor	Sterilization and nutrient balancing	*Serratia marcescens*	Prodigiosin	SmF	~3–5 g/L prodigiosin	Solvent extraction (methanol)	Laboratory	Antimicrobial, anticancer compounds	Pigment instability; regulatory concerns	Elkenawy et al., 2017
Fruit peels (orange, pomegranate)	Drying and hydrolysis	*Rhodotorula mucilaginosa*	Carotenoids	SmF	~15–20 mg/L	Solvent extraction	Laboratory	Functional foods, cosmetics	Seasonal substrate variability	Taskin et al., 2011
Vegetable waste hydrolysate	Enzymatic hydrolysis	*Monascus ruber*	Red pigments	SmF	Comparable to synthetic medium	Ethanol extraction	Laboratory	Natural food colorants	Inhibitor formation during hydrolysis	Carvalho et al., 2003
Brewery spent grain	Milling and enzymatic saccharification	*Monascus purpureus*	Monascus pigments	SSF	Moderate–high pigment productivity	Solvent extraction	Laboratory/Pilot	Food and textile dye	Complex lignocellulosic structure	Mussagy et al., 2019
Sugarcane bagasse hydrolysate	Dilute acid hydrolysis	*Talaromyces* spp.	Carotenoids	SSF	Increased pigment synthesis relative to untreated substrate	Solvent extraction	Laboratory	Textile dyes, antioxidants	Pretreatment cost; inhibitor formation	Chaiyasut et al., 2016
Rice straw hydrolysate	Acid pretreatment + detoxification	*Serratia marcescens*	Prodigiosin	SmF	Improved yield relative to glucose medium	Solvent extraction	Laboratory	Biomedical and antimicrobial applications	Hydrolysate toxicity	Ahmed et al. (2021)
Fishery waste (shrimp shell hydrolysate)	Enzymatic hydrolysis	Marine bacteria (*Pseudoalteromonas* spp.)	Carotenoids	SmF	Moderate pigment yield	Solvent extraction	Laboratory	Cosmetics, pharmaceuticals	Salt sensitivity; scale-up limitations	Wang et al., 2022
Food waste mixed hydrolysate	Thermal pretreatment + enzymatic hydrolysis	*Talaromyces albobiverticillius* + *Trichoderma reesei*	Red fungal pigments	Co-culture SSF	Significantly improved yield vs. monoculture	Solvent extraction	Laboratory	Textile dyes, food colorants	Co-culture stability; reproducibility	Zhang et al., 2023
Municipal wastewater	Filtration and nutrient adjustment	*Arthrospira platensis*	Phycocyanin	Photobioreactor cultivation	Variable productivity depending on nutrient load	Membrane filtration	Pilot scale	Nutraceuticals, fluorescent probes	Contamination risk; regulatory approval	Pagels et al., 2022

### 6.1 Agro-industrial residues: molasses, whey, rice bran, and corn steep liquor

Agro-industrial residues generated during food and crop processing have been widely investigated as economical substrates for microbial pigment production. These by-products are available in large quantities and possess nutrient profiles comparable to conventional media, making them suitable for industrial fermentation processes (Lopes and Ligabue-Braun, 2021). Molasses, a by-product of the sugar industry, is rich in sucrose, glucose, fructose, and essential minerals. Its high carbon content has been shown to effectively support pigment biosynthesis by microorganisms such as *Rhodotorula*, *Serratia*, and *Monascus*, leading to enhanced production of carotenoids and prodigiosin. The use of molasses as a primary carbon source has consistently demonstrated reduced medium costs without compromising pigment yield (Zhang et al., 2021). Whey, the lactose- and protein-rich effluent from cheese manufacturing, has also emerged as a promising substrate for microbial pigment production. Due to its high organic load, whey supports robust microbial growth and has been successfully utilized for the production of red pigments by *Monascus purpureus* under submerged fermentation (Poonia and Pandey, 2023). Importantly, whey-based fermentation offers an integrated approach to pigment production and dairy waste management. Rice bran, an abundant by-product of rice milling, contains fermentable carbohydrates, lipids, amino acids, and micronutrients that promote pigment accumulation in fungi and yeasts. Its oil fraction and bioactive compounds have been reported to enhance pigment biosynthesis, particularly under solid-state fermentation conditions (Nidhishree et al., 2024). Corn steep liquor, a liquid residue from corn wet-milling, provides a balanced source of carbon, nitrogen, vitamins, and trace elements. Its application as an inexpensive nitrogen-rich supplement has been shown to enhance microbial growth and pigment production while significantly reducing reliance on costly peptone or yeast extract (Chang et al., 2025). Collectively, agro-industrial residues such as molasses, whey, rice bran, and corn steep liquor represent sustainable and economically viable substrates for microbial pigment production. These substrates continue to gain attention because of their rich nutrient composition and suitability in large-scale fermentation processes.

### 6.2 Food processing wastes (fruit peels, vegetable residues, and brewery waste)

Food processing industries generate substantial quantities of organic residues, particularly fruit peels, vegetable trimmings, and brewery by-products, which are increasingly recognized as valuable substrates for microbial pigment production. These wastes are rich in fermentable carbohydrates, dietary fibers, vitamins, minerals, and phenolic compounds that support microbial growth and secondary metabolite biosynthesis (Bezirhan Arikan et al., 2020). Fruit peels such as apple and citrus wastes contain soluble sugars, organic acids, and micronutrients that promote carotenoid and polyketide pigment production by bacteria and yeasts, including *Rhodotorula*, *Serratia*, and *Monascus* species (Afroz Toma et al., 2023).

Vegetable residues provide similar fermentable biomass fractions, along with antioxidant precursors that may enhance pigment stability and bioactivity. In parallel, brewery spent grains and surplus yeast generated during beer production are protein-rich and mineral-dense substrates that effectively support microbial biomass formation and pigment synthesis under both submerged and solid-state fermentation systems (Adil et al., 2025). These food-derived wastes have been successfully employed for the production of high-value pigments such as Monascus red pigments, carotenoids, and prodigiosin, highlighting their versatility and suitability for scalable, low-cost bioprocesses.

### 6.3 Lignocellulosic biomass and forestry residues

Lignocellulosic biomass, comprising cellulose, hemicellulose, and lignin, represents one of the most abundant renewable organic resources generated from agricultural residues, crop stalks, straw, bagasse, and forestry by-products. Despite their abundance, these materials are inherently recalcitrant to direct microbial utilization due to the complex and rigid structure of plant cell walls (Oliveira et al., 2024). Consequently, pretreatment processes such as dilute acid hydrolysis, enzymatic saccharification, or mild physical treatments are required to release fermentable sugars. Once processed, lignocellulosic hydrolysates have been effectively utilized as carbon sources for microbial pigment production. For instance, rice straw hydrolysates have supported prodigiosin production by *Serratia* species, while corncob and bagasse hydrolysates have been employed for carotenoid biosynthesis by fungal strains such as *Talaromyces* (Venil et al., 2020a). The integration of pigment production into lignocellulosic biorefineries not only diversifies the product portfolio but also enhances overall process sustainability by converting low-value residues into high-value bioproducts.

### 6.4 Marine and fishery wastes

Marine and fishery processing activities generate large quantities of waste materials, including shells, fish offal, viscera, and nutrient-rich effluents, which remain underutilized despite their high biotechnological potential. These wastes are rich in proteins, chitin, lipids, and minerals that can support the growth of marine-adapted microorganisms capable of pigment biosynthesis (Muñoz-Miranda and Iñiguez-Moreno, 2023). Marine bacteria and cyanobacteria cultivated on fishery waste streams have been reported to produce carotenoids, phycobiliproteins, and other bioactive pigments with antioxidant and antimicrobial properties (Nurkolis, 2025). Notably, pigments derived from marine waste-based systems often exhibit enhanced functional properties, attributed to the unique metabolic adaptations of marine microorganisms. Thus, the use of marine and fishery wastes for pigment production offers an additional resource for the production of functional microbial pigments by reducing seafood processing waste.

### 6.5 Industrial and municipal organic wastes

Industrial organic effluents, such as brewery wastewater and sugar processing effluents, along with municipal food waste streams, represent complex but nutrient-rich substrates for microbial cultivation (Maria et al., 2023). These wastes contain fermentable carbon sources, nitrogenous compounds, and trace elements that can support the growth of heterotrophic microbes, cyanobacteria, and microalgae capable of pigment production. Several studies have demonstrated the successful cultivation of cyanobacteria and microalgae in industrial and municipal wastewaters for the production of phycobiliproteins and carotenoids, simultaneously achieving nutrient removal and wastewater remediation (Usmani et al., 2020). Despite challenges such as variable composition, potential toxic inhibitors, and contamination risks, advances in pretreatment technologies, adaptive laboratory evolution, and microbial consortia engineering are expanding the feasibility of using industrial and municipal wastes as reliable fermentation substrates. Consequently, these waste streams represent promising alternative substrates for pigment producing microorganisms.

## 7 Pretreatment and valorization strategies for waste substrates

**Figure 3:**
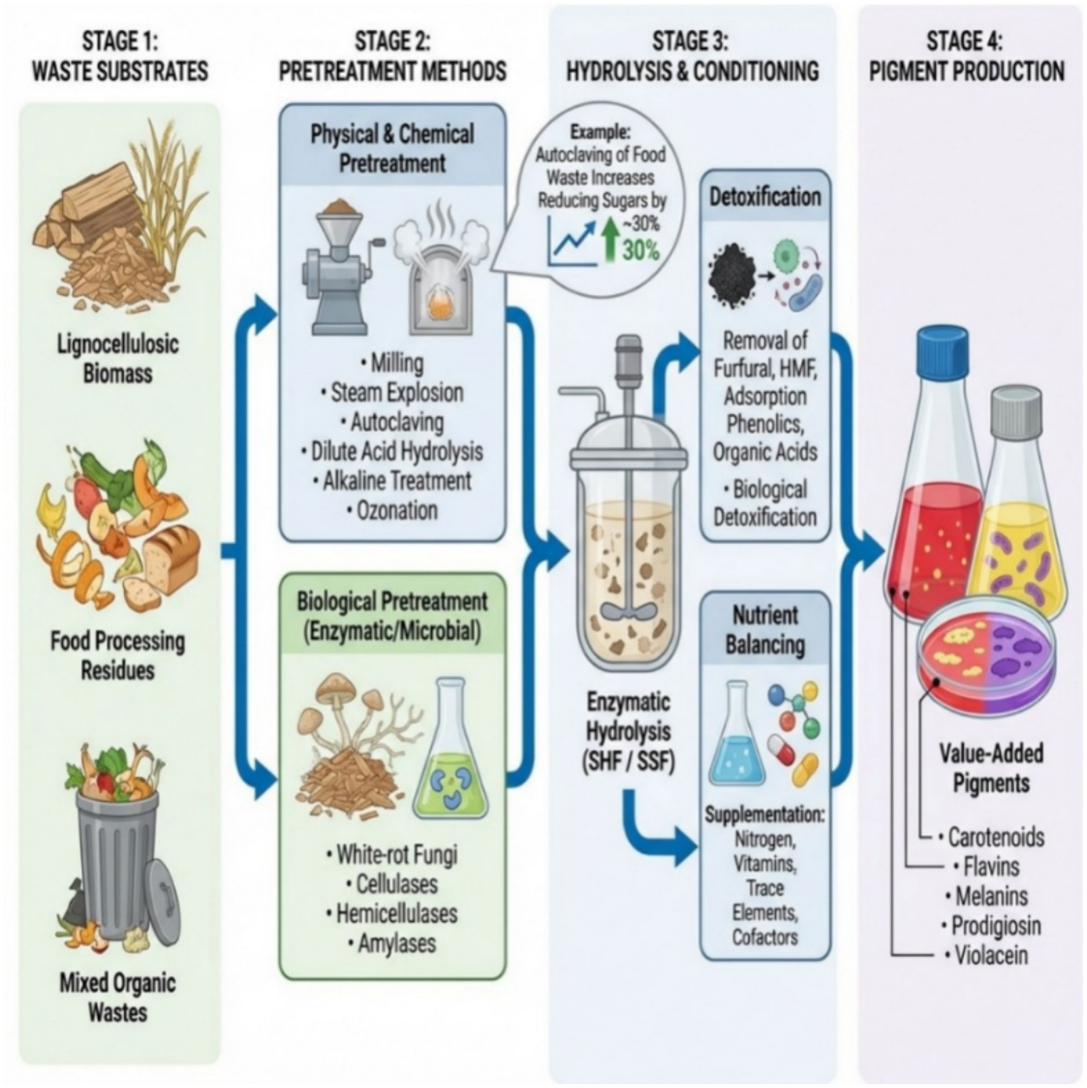
Pretreatment and valorization pathways for converting waste into fermentable substrates.

The effective microbial conversion of complex organic wastes into value-added pigments depends critically on appropriate pretreatment and valorization strategies. Waste substrates such as lignocellulosic biomass, food processing residues, and mixed organic wastes are structurally heterogeneous and often contain recalcitrant polymers, inhibitory compounds, and imbalanced nutrient profiles that limit microbial accessibility to fermentable carbon and essential growth factors (Lopes and Ligabue-Braun, 2021). Consequently, pretreatment and downstream conditioning are indispensable for enhancing substrate digestibility, mitigating inhibitory effects, and optimizing nutrient availability to achieve high pigment yields (Figure 3).

### 7.1 Physical and chemical pretreatment methods

Physical and chemical pretreatment approaches are widely employed to disrupt complex biomass architectures, increase surface area, and liberate fermentable sugars that can be readily assimilated by pigment-producing microorganisms. Physical methods such as milling, grinding, steam explosion, and hydrothermal treatment mechanically or thermally alter biomass structure, thereby enhancing enzymatic accessibility and microbial utilization (Usmani et al., 2020; Ahmed et al., 2025). Thermal treatments, including autoclaving and steam explosion, weaken hydrogen bonding within lignocellulosic matrices and promote the release of reducing sugars, while also contributing to microbial decontamination. For example, autoclave pretreatment of food waste has been reported to increase reducing sugar availability by approximately 30%, thereby improving heterotrophic growth potential for *Chlorella vulgaris* cultivation (Marques et al., 2024). Chemical pretreatment methods such as dilute acid hydrolysis, alkaline treatment, ozonation, and oxidative delignification further enhance biomass digestibility by solubilizing hemicellulose and partially removing lignin. Dilute acid pretreatment effectively hydrolyzes hemicellulose fractions into monomeric sugars, whereas alkaline pretreatment preferentially removes lignin and improves enzymatic access to cellulose. Despite their effectiveness, chemical pretreatments may generate inhibitory by-products, including furfural, hydroxymethylfurfural (HMF), organic acids, and phenolic compounds. Additionally, the use of harsh chemicals raises environmental and economic concerns, necessitating careful optimization or integration with downstream detoxification and biological processes (Marques et al., 2024) (Figure 3).

### 7.2 Enzymatic and microbial hydrolysis

Enzymatic hydrolysis represents a more selective and environmentally benign alternative to harsh chemical pretreatment. This approach employs hydrolytic enzymes such as cellulases, hemicellulases, and amylases to convert complex polysaccharides into fermentable monosaccharides under mild operating conditions, minimizing the formation of toxic by-products (Ramzan et al., 2025). For recalcitrant wastes, enzymatic hydrolysis is typically preceded by physical or chemical pretreatment to expose cellulose and hemicellulose to enzymatic attack. Process configurations commonly include separate hydrolysis and fermentation (SHF), in which saccharification is completed prior to microbial cultivation, and simultaneous saccharification and fermentation (SSF), where hydrolysis and fermentation occur concurrently to reduce processing time and alleviate end-product inhibition (Ramzan et al., 2025). In addition, microbial hydrolysis using cellulolytic and ligninolytic microorganisms particularly white-rot fungi offers a biological pretreatment strategy capable of selective lignin degradation and *in situ* enzyme production. Such organisms improve biomass fermentability over extended incubation periods without the need for hazardous chemicals (Wu et al., 2022). Nevertheless, enzymatic and microbial hydrolysis approaches face limitations, including enzyme costs, slow reaction kinetics, and substrate or product inhibition. Current research efforts are therefore focused on integrated enzyme production systems, co-culture strategies, and on-site enzyme generation to enhance economic feasibility and process scalability (Figure 3).

### 7.3 Detoxification and nutrient balancing

Pretreatment processes frequently result in the accumulation of inhibitory compounds such as furans (furfural and HMF), phenolics, organic acids, and other degradation products that negatively affect microbial growth and pigment biosynthesis. These inhibitors may disrupt membrane integrity, inhibit key metabolic enzymes, induce oxidative stress, and alter intracellular redox balance, ultimately reducing fermentation efficiency (Liu et al., 2020; Marques et al., 2024). Detoxification strategies are therefore essential to improve hydrolysate compatibility with pigment-producing microbes. Physical approaches such as over liming and activated carbon adsorption remove inhibitory molecules, while biological detoxification employs specialized microorganisms capable of metabolizing toxic intermediates. Chemical conditioning methods, including pH adjustment and precipitation, have also been applied to neutralize inhibitory effects (Liu et al., 2020). In parallel, nutrient balancing plays a critical role in sustaining microbial metabolism and directing carbon flux toward pigment biosynthesis. Waste substrates often exhibit variable carbon-to-nitrogen ratios and inconsistent micronutrient availability. Supplementation with nitrogen sources, vitamins, trace elements, or cofactors can correct nutritional deficiencies and stabilize pigment production performance (Merchant and Helmann, 2012). Integrated valorization strategies increasingly combine detoxification and nutrient conditioning, for example, through biological pretreatment systems that detoxify while simultaneously releasing bioavailable nutrients into the hydrolysate (Figure 3).

## 8 Microbial fermentation strategies in circular pigment production

Microbial fermentation constitutes the core biotechnological platform for converting waste-derived substrates into value-added pigments. The selection and design of fermentation strategies strongly influence substrate utilization efficiency, pigment yield, metabolic robustness, and overall process scalability. Within circular bioprocessing frameworks, conventional fermentation systems are being increasingly adapted to accommodate heterogeneous waste streams while minimizing environmental impact, energy input, and production costs (Devi et al., 2024).

### 8.1 Submerged fermentation

Submerged fermentation (SmF), also referred to as liquid-state fermentation, involves the cultivation of microorganisms in a fully liquid medium where nutrients and cells are uniformly suspended. SmF systems enable efficient mass and heat transfer and allow precise control over key physicochemical parameters, including pH, temperature, dissolved oxygen, aeration, and agitation. Such control is particularly advantageous when fermenting waste-derived hydrolysates with variable composition, as process parameters can be adjusted in real time to maintain metabolic stability (Devi et al., 2025b). Due to its high reproducibility and compatibility with stirred-tank and airlift bioreactors, SmF remains the dominant strategy for industrial pigment production. Continuous monitoring and automation facilitate scale-up and product standardization, which are critical for commercial deployment. Optimization of SmF processes increasingly relies on statistical and modeling tools such as Response Surface Methodology (RSM) and Box–Behnken design to systematically evaluate interactions among environmental parameters and substrate concentrations. Such approaches have been successfully applied to maximize pigment production, including prodigiosin biosynthesis from lignocellulosic and agro-waste hydrolysates (Pérez-Jiménez et al., 2025). Despite these advantages, SmF faces limitations related to oxygen transfer constraints at high cell densities and significant energy demands for agitation and aeration, particularly during large-scale operation. Nevertheless, its operational flexibility and scalability continue to make SmF the preferred platform for commercial microbial pigment fermentation.

### 8.2 Solid-state fermentation

Solid-state fermentation (SSF) involves microbial growth on moist solid substrates in the absence of free-flowing water, with the solid matrix serving as both nutrient source and physical support. SSF closely mimics the natural ecological niches of filamentous fungi and certain yeasts, making it especially suitable for pigment production from agro-industrial residues such as rice bran, wheat bran, husks, and other fibrous wastes (Pérez-Jiménez et al., 2025). SSF offers several advantages in circular bioprocessing, including reduced water consumption, lower wastewater generation, and often higher product concentration per unit substrate mass. These attributes can significantly improve process economics when solid wastes are used directly with minimal pretreatment. Filamentous fungi, such as *Aspergillus*, *Monascus*, and *Talaromyces* species, exhibit invasive hyphal growth that enhances substrate penetration and metabolite synthesis, frequently resulting in higher pigment titers compared to submerged systems (Perwez and Al Asheh, 2025). However, SSF presents technical challenges related to heterogeneous temperature, moisture, and oxygen gradients within the solid matrix. These limitations complicate real-time monitoring, process control, and scale-up, particularly in large packed-bed or tray fermenters. As a result, while SSF is attractive for waste valorization at small to medium scales, its industrial implementation remains less standardized than SmF.

### 8.3 Co-culture and mixed microbial systems

Co-culture and mixed microbial fermentation systems have gained increasing attention as strategies to enhance pigment production from complex waste substrates. In natural ecosystems, microorganisms function within communities where metabolic cooperation, cross-feeding, and signaling interactions stimulate biosynthetic pathways that may remain inactive in monocultures. Translating these interactions into engineered fermentation systems offers opportunities to improve substrate conversion efficiency and pigment yield (Rosero-Chasoy et al., 2021). Under SSF conditions, co-cultivation of complementary fungal species has been shown to enhance pigment production relative to single-strain systems. For example, co-culture of *Talaromyces albobiverticillius* and *Trichoderma reesei* on food waste substrates resulted in significantly higher pigment yields, demonstrating how synergistic enzyme production and metabolic cooperation can improve waste valorization (Troiano et al., 2022). In SmF systems, co-culture approaches can be designed to distribute metabolic tasks, with one microorganism hydrolyzing complex polymers and another channeling metabolic flux toward pigment biosynthesis. Engineered microbial consortia have also been explored to facilitate extracellular pigment secretion and broaden pigment diversity (Troiano et al., 2022). Despite their promise, co-culture systems require careful optimization of inoculum ratios, timing of inoculation, and environmental conditions to avoid competitive exclusion or dominance by a single species. Maintaining stable community dynamics during scale-up remains a major challenge, limiting current applications primarily to laboratory and pilot scales.

### 8.4 Process optimization and scale-up challenges

Across all fermentation strategies, systematic process optimization is essential to achieve high pigment productivity, reproducibility, and economic viability. Optimization efforts encompass medium composition, waste substrate loading, environmental parameters, and operational modes such as batch, fed-batch, and continuous fermentation (Venkatachalam, 2024). Advanced statistical tools, including Design of Experiments (DoE) and RSM, enable efficient identification of optimal conditions while reducing experimental complexity, particularly when working with variable waste-derived feedstocks. Scale-up introduces additional challenges that differ across fermentation platforms. SmF scale-up is comparatively well-established due to homogeneous liquid conditions and availability of standardized bioreactors; however, maintaining adequate oxygen transfer, mixing, and heat dissipation at large volumes remains technically demanding (Mohd Said, 2023). SSF scale-up is inherently more complex due to mass and heat transfer limitations within solid matrices, which are exacerbated by the heterogeneity of raw waste substrates. Co-culture and mixed microbial systems further complicate scale-up, as stable maintenance of species interactions requires precise control over microbial population dynamics (Bamidele et al., 2025). Emerging strategies such as continuous fermentation, immobilized cell systems, and novel bioreactor designs tailored for multi-organism cultures offer potential solutions but require further investigation to enable reliable industrial implementation.

## 9 Metabolic engineering and synthetic biology approaches

**Figure 4:**
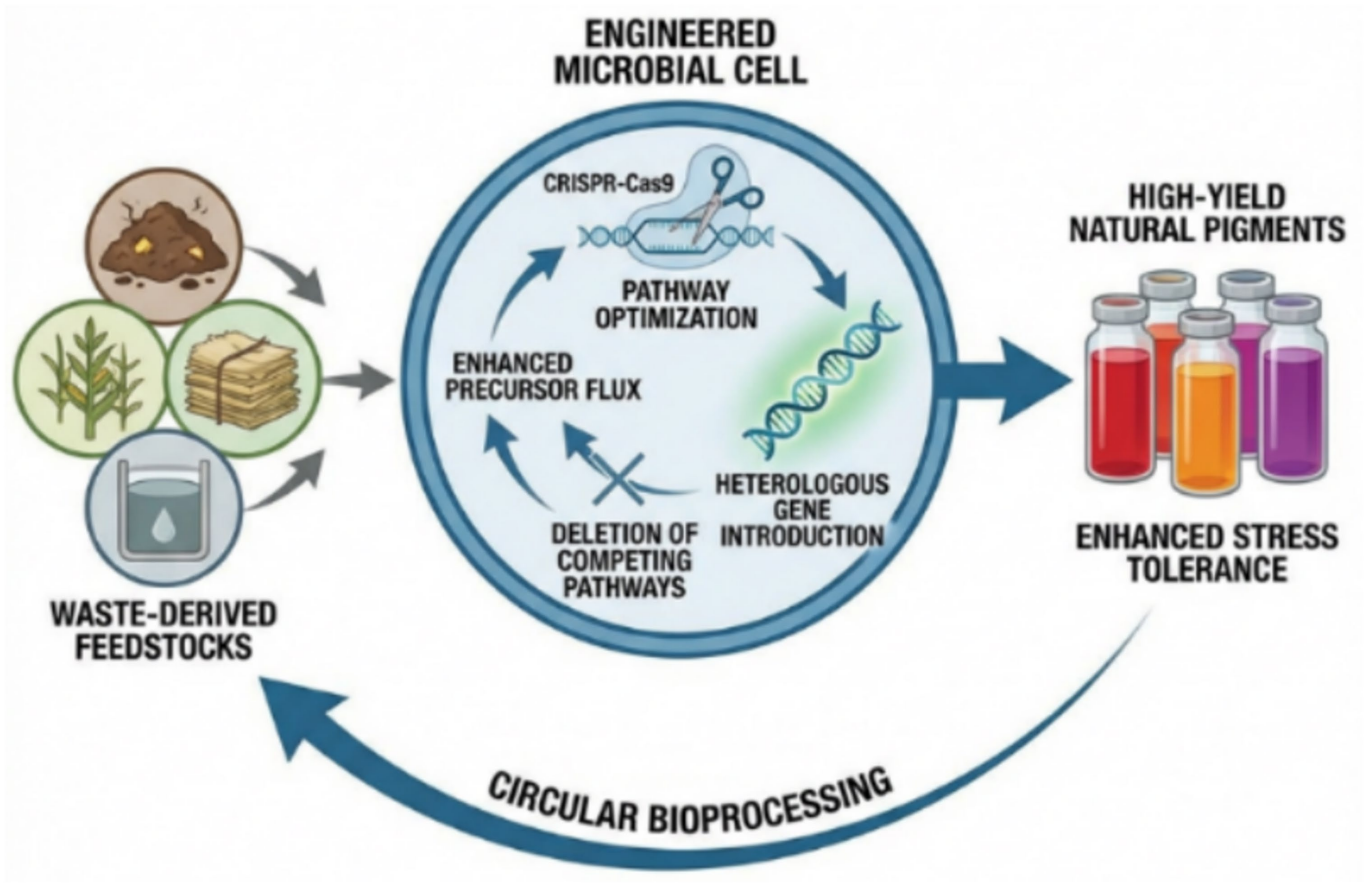
Metabolic engineering strategies for improved microbial pigment biosynthesis.

Metabolic engineering and synthetic biology have emerged as transformative tools for enhancing microbial pigment production, particularly in circular bioprocesses that rely on waste-derived feedstocks. Unlike conventional strain screening or fermentation optimization alone, these approaches enable the rational redesign of cellular metabolism to increase flux toward target pigments, broaden substrate utilization, and improve tolerance to environmental stressors commonly associated with heterogeneous waste streams. Such capabilities are essential for achieving robust, high-yield pigment production under economically and environmentally sustainable conditions (Figure 4).

### 9.1 Engineering microbes for enhanced pigment yield

Engineering microorganisms for improved pigment production typically involves systematic modification of native metabolic pathways through gene overexpression, deletion of competing pathways, and introduction of heterologous biosynthetic modules. These strategies aim to increase the availability of key precursors, reduce metabolic bottlenecks, and enhance catalytic efficiency within pigment biosynthetic pathways (Usmani et al., 2020). Carotenoid biosynthesis provides a well-established example of pathway engineering for pigment overproduction. In *Escherichia coli* and *Saccharomyces cerevisiae*, the assembly and optimization of enzymes involved in the mevalonate or methylerythritol phosphate pathways such as isopentenyl pyrophosphate isomerase (IDI) and geranylgeranyl diphosphate synthase (CrtE) have enabled more efficient channeling of metabolic flux toward carotenoid synthesis (Ravi Gopal and Wang, 2025). Modular pathway designs and enzyme colocalization strategies have been shown to significantly increase lycopene yields, in some cases exceeding 50% improvements over non-engineered strains. Heterologous expression of pigment gene clusters has further expanded the biosynthetic capabilities of well-characterized microbial hosts. For example, the introduction of *β*-carotene hydroxylase (crtZ) and β-carotene ketolase (crtW) genes into *S. cerevisiae* has enabled efficient astaxanthin production through optimized, multi-step biosynthetic cascades. Beyond yield enhancement, metabolic engineering allows the tailoring of pigment properties, including color intensity, stability, and bioactivity, thereby expanding their applicability in food, cosmetic, and pharmaceutical industries (Nogueira et al., 2019; Figure 4).

### 9.2 Utilization of heterogeneous waste carbon sources

Waste-derived carbon sources offer significant economic and environmental advantages for pigment bioproduction but pose metabolic challenges due to their complex and variable composition. Metabolic engineering enables microorganisms to efficiently assimilate such heterogeneous substrates by expanding substrate uptake systems and catabolic pathways for lignocellulosic sugars, organic acids, and mixed carbohydrate streams (Tuli et al., 2015). Advances in systems biology and multi-omics analyses have facilitated the identification of metabolic bottlenecks that arise when microbes are cultivated on non-conventional carbon sources. Engineered cell factories increasingly incorporate tailored modules for transport and catabolism of waste-derived substrates, allowing robust growth and pigment biosynthesis on feedstocks that are poorly utilized by native strains (Jiang et al., 2025). In metabolically versatile chassis organisms such as *Pseudomonas putida*, genome-scale metabolic models have guided the introduction of pathways that tightly couple growth to pigment formation, thereby minimizing by-product accumulation and improving carbon conversion efficiency (Weimer et al., 2020). Additionally, engineered strains can be designed to tolerate inhibitory compounds commonly present in lignocellulosic hydrolysates and food waste streams. Enhancing stress resistance and detoxification capacity is particularly important in circular bioprocessing, where feedstock variability and inhibitor presence are difficult to eliminate (Adebule et al., 2026; Figure 4).

### 9.3 CRISPR-based pathway optimization for circular bioprocesses

The advent of CRISPR–Cas genome editing technologies has revolutionized metabolic engineering by enabling precise, rapid, and multiplexed genetic modifications. In the context of microbial pigment production, CRISPR-based tools have been employed to delete competing metabolic pathways, upregulate rate-limiting enzymes, and fine-tune regulatory networks to improve pigment yield and stability (Seralathan et al., 2025). CRISPR-mediated gene knockouts redirect metabolic flux toward pigment biosynthesis by eliminating competing precursor sinks, while targeted editing of transcriptional regulators enables improved balancing of cellular resources between growth and product formation. Such control is particularly valuable in waste-based fermentations, where nutrient availability and metabolic fluxes can fluctuate over time. Moreover, CRISPR interference (CRISPRi) and CRISPR activation (CRISPRa) systems allow dynamic and reversible modulation of gene expression without permanent genomic alterations, providing metabolic flexibility under variable process conditions (Seralathan et al., 2025). Emerging applications of CRISPR in microalgae further highlight its potential for circular pigment production. Editing of photosynthetic efficiency, carbon fixation pathways, and pigment biosynthesis genes has been shown to enhance pigment accumulation by improving light energy utilization and carbon assimilation. These advances are especially relevant for large-scale phototrophic systems that integrate waste gas streams or wastewater as nutrient sources, positioning CRISPR-enabled microalgae as promising platforms for sustainable pigment biomanufacturing (Seralathan et al., 2025; Figure 4).

## 10 Downstream processing and product recovery

While upstream fermentation governs microbial growth and pigment biosynthesis, downstream processing ultimately determines the feasibility, sustainability, and commercial success of microbial pigment production. In waste-derived circular bioprocesses, downstream operations including extraction, purification, formulation, and stabilization are critical for converting fermented biomass into market-ready pigments that meet industrial quality and safety standards (Lyu et al., 2022). Notably, downstream processing can account for nearly 60–70% of the total production cost, emphasizing the need for efficient, low-energy, and environmentally benign recovery strategies. Consequently, innovations that reduce solvent usage, processing time, and waste generation are central to advancing sustainable microbial pigment manufacturing.

### 10.1 Extraction and purification of pigments from waste-based fermentations

Microbial pigments exhibit diverse physicochemical properties, ranging from hydrophobic intracellular carotenoids and melanins to extracellular, water-soluble pigments such as phycobiliproteins. This diversity necessitates tailored extraction and purification strategies depending on pigment class and cellular localization. Conventional extraction approaches commonly employ organic solvents including ethanol, acetone, methanol, hexane, or tetrahydrofuran to solubilize pigments effectively (Al-Monofy et al., 2025). Despite their efficiency, these solvents raise concerns related to toxicity, flammability, environmental persistence, and regulatory compliance, particularly for food and cosmetic applications. For intracellular pigments, cell disruption is a prerequisite to solvent extraction. Mechanical methods such as bead milling and high-pressure homogenization are widely used but are energy-intensive and may induce thermal degradation. Emerging non-thermal techniques including pulsed electric fields (PEF) and microwave-assisted extraction (MAE)have gained attention for enhancing cell permeability and mass transfer while preserving pigment integrity (Zhao et al., 2024). Following extraction, purification is commonly achieved through chromatographic techniques such as column chromatography and preparative thin-layer chromatography, while high-performance liquid chromatography (HPLC) coupled with mass spectrometry or nuclear magnetic resonance (NMR) is routinely employed for pigment identification and purity assessment. Additionally, adsorption resins and membrane-based separations have emerged as scalable alternatives, enabling selective pigment recovery directly from fermentation broths and significantly reducing solvent consumption (Mahato et al., 2019).

### 10.2 Pigment identification and characterization

The accurate characterization of microbial pigments generally requires the application of complementary analytical tools, as color-based observations and UV–vis absorbance measurements alone often inadequate for definitive pigment identification. Chromatographic techniques such as thin-layer chromatography (TLC), high-performance liquid chromatography (HPLC), column chromatography, and are extensively employed to isolate and purify of microbial pigments (Valenzuela-Gloria et al., 2021; Rajendran et al., 2023). UV–Vis spectroscopy is commonly employed to assess the absorption properties of pigments, whereas FTIR analysis provides information regarding the functional groups associated with pigment molecules (Valenzuela-Gloria et al., 2021). Techniques including LC–MS and LC–MS/MS are widely used to determine the molecular profiling of pigments, whereas NMR spectroscopy facilitates detailed structural analysis (Valenzuela-Gloria et al., 2021; Rajendran et al., 2023). The combination of chromatographic purification and advanced spectroscopic and spectrometric techniques enhances the accuracy of pigment identification and characterizing their molecular properties (Valenzuela-Gloria et al., 2021; Rajendran et al., 2023).

### 10.3 Green solvents and sustainable recovery methods

In alignment with green chemistry and circular economy principles, downstream innovations increasingly emphasize sustainable extraction technologies that minimize environmental impact while maintaining high recovery efficiency. Alternative solvent systems such as ionic liquids, deep eutectic solvents (DESs), and aqueous surfactant-based systems have demonstrated strong potential for extracting microbial pigments with high selectivity and stability. These solvents offer advantages including low vapor pressure, tunable polarity, and recyclability, making them environmentally preferable to conventional petroleum-derived solvents (Pandit and Saha, 2025). Advanced extraction technologies such as ultrasound-assisted extraction (UAE), enzyme-assisted extraction (EAE), and pressurized liquid extraction (PLE) further enhance pigment recovery while reducing processing time, solvent volume, and energy input. UAE facilitates pigment release through acoustic cavitation, while PLE improves mass transfer under controlled pressure and temperature conditions, preserving heat-sensitive bioactive pigments (Morón-Ortiz et al., 2024). Biological extraction strategies, particularly enzymatic hydrolysis of microbial cell walls, offer gentle and highly selective pigment recovery under mild conditions. Enzymes such as glucanases, glycosidases, and proteases improve pigment accessibility while minimizing oxidative and thermal degradation, making them particularly attractive for high-value pigment applications (Wang et al., 2025). Furthermore, integration of adsorbent resins, immobilized matrices, and *in situ* product recovery systems allows direct pigment capture during or immediately after fermentation (Evans and Wang, 1984). Such integrated approaches reduce downstream complexity, facilitate solvent recycling, and improve overall process sustainability.

### 10.4 Quality, safety, and regulatory considerations

Downstream processing is intrinsically linked to quality assurance and regulatory compliance, particularly for pigments intended for food, nutraceutical, cosmetic, and pharmaceutical applications. Regulatory agencies such as the U. S. Food and Drug Administration (FDA) and the European Food Safety Authority (EFSA) require comprehensive safety evaluations, including assessments of toxicity, stability, purity, and residual solvent levels. Microbial pigments must also demonstrate batch-to-batch consistency and absence of harmful contaminants such as endotoxins or mycotoxins (Rodríguez-Sifuentes et al., 2021). For food-grade applications, regulatory approval depends on evaluations of acceptable daily intake, interactions with food matrices, and extraction solvent safety. Consequently, the use of water and food-grade solvents such as ethanol is strongly preferred, as non-approved organic solvents may limit regulatory acceptance and consumer trust (Molina et al., 2023). Pigment stability represents another critical quality parameter, as many natural pigments are sensitive to light, oxygen, temperature, and pH. Degradation during processing and storage can compromise color intensity and functional properties. Formulation strategies such as microencapsulation, co-pigmentation, and antioxidant incorporation have proven effective in enhancing pigment stability, shelf life, and performance in final applications (Masyita et al., 2025). Finally, compliance with environmental regulations governing solvent emissions, wastewater discharge, and solid waste management is essential, particularly in circular bioprocesses that aim to minimize ecological footprints. Sustainable downstream strategies must therefore integrate solvent recovery, energy-efficient unit operations, and life cycle–oriented process design to ensure both regulatory compliance and environmental stewardship (Table 3).

**Table 3:** Downstream processing techniques and recovery efficiencies for microbial pigments.

Downstream processing step	Technique	Principle	Pigments recovered	Typical recovery efficiency (%)	Advantages	Limitations
Biomass separation	Centrifugation	Separation based on density differences	Carotenoids, prodigiosin, violacein, melanin	85–98	Fast, efficient, widely used	High energy consumption, costly at industrial scale
Biomass separation	Filtration (membrane/vacuum)	Size-based separation using porous membranes	Phycocyanin, riboflavin, carotenoids	80–95	Simple, scalable	Membrane fouling, slower than centrifugation
Cell disruption	Ultrasonication	Acoustic cavitation breaks cell walls	Carotenoids, violacein, prodigiosin	75–95	Efficient, rapid	Heat generation may degrade pigments
Cell disruption	Bead milling	Mechanical shear force disrupts cells	Intracellular pigments (carotenoids, prodigiosin)	85–98	Suitable for large-scale processing	Equipment wear, high energy use
Cell disruption	Enzymatic lysis	Enzymes degrade cell wall components	Phycobiliproteins, carotenoids	80–95	Mild conditions, preserves pigment stability	Expensive enzymes, longer processing time
Solvent extraction	Organic solvents (methanol, ethanol, acetone)	Solubilization based on pigment polarity	Carotenoids, prodigiosin, violacein	80–99	High recovery efficiency, simple	Solvent toxicity, environmental concerns
Solvent extraction	Supercritical CO₂ extraction	Solvent extraction using supercritical fluids	Carotenoids, astaxanthin	85–98	Eco-friendly, high purity	Expensive equipment, high pressure required
Purification	Column chromatography	Separation based on polarity and adsorption	Violacein, prodigiosin, carotenoids	70–95	High purity	Time-consuming, costly
Purification	Membrane filtration (ultrafiltration)	Molecular size-based separation	Phycocyanin, phycoerythrin	75–90	Scalable, no solvents	Membrane fouling
Purification	Aqueous two-phase system (ATPS)	Partitioning between immiscible aqueous phases	Phycobiliproteins, carotenoids	80–95	Eco-friendly, scalable	Optimization required
Drying and stabilization	Spray drying	Rapid solvent evaporation using hot air	Phycocyanin, carotenoids	70–90	Industrially scalable	Heat sensitivity of pigments
Drying and stabilization	Freeze drying	Sublimation under low temperature	Sensitive pigments (phycocyanin, violacein)	85–98	Preserves pigment stability	Expensive, slow

## 11 Applications of waste-derived microbial pigments

Microbial pigments produced using agro-industrial and other waste substrates represent a sustainable and economically viable alternative to synthetic colorants. These pigments, including carotenoids, prodigiosin, violacein, melanins, and riboflavin, exhibit diverse bioactive properties such as antioxidants, antimicrobial, anticancer, and anti-inflammatory activities, making them highly valuable across food, pharmaceutical, cosmetic, and industrial sectors. Waste-based pigment production supports circular bioeconomy principles by converting low-value residues into high-value bioproducts while reducing environmental pollution and production costs (Grewal et al., 2022; Lopes and Ligabue-Braun, 2021).

### 11.1 Food and nutraceutical industries

Waste-derived microbial pigments have gained significant attention as natural colorants and functional ingredients in food and nutraceutical applications. Pigments such as *β*-carotene, lycopene, riboflavin, and astaxanthin are already used commercially in beverages, dairy products, confectionery, and processed foods due to their excellent coloring properties and safety profile (Grewal et al., 2022). These pigments also possess antioxidant and antimicrobial activities, which enhance food preservation, improve shelf life, and contribute to nutritional value. In nutraceutical formulations, microbial pigments provide additional health benefits such as anti-inflammatory, immune-modulatory, and metabolic protective effects. Carotenoid pigments, for example, function as provitamin A compounds and help prevent oxidative stress-related diseases, cardiovascular disorders, and degenerative conditions (Chettri et al., 2025). The use of agro-industrial waste substrates such as molasses, whey, and fruit peels for pigment production further enhances economic feasibility and sustainability while producing food-grade pigments suitable for dietary supplements and functional foods (Panesar et al., 2015).

### 11.2 Pharmaceuticals and biomedical applications

Microbial pigments derived from waste substrates exhibit remarkable pharmacological properties, making them promising candidates for pharmaceutical and biomedical applications. Several pigments, including prodigiosin, violacein, carotenoids, and melanins, demonstrate antimicrobial, antiviral, anticancer, anti-inflammatory, and antioxidant activities (Grewal et al., 2022). For example, prodigiosin produced by actinomycetes has shown gastroprotective and antibacterial effects, while microbial astaxanthin exhibits cytotoxic activity against cancer cell lines and protects against oxidative stress (Grewal et al., 2022). In addition, microbial pigments are being explored for biomedical applications such as wound healing, drug delivery, diagnostic imaging, and tissue engineering due to their biocompatibility and bioactivity. Their ability to act as natural antioxidants and free radical scavengers makes them useful in preventing cellular damage and managing chronic diseases (Bałdowska-Witos et al., 2023). Furthermore, pigment-based biomolecules derived from waste fermentation systems provide a sustainable platform for producing therapeutic compounds with reduced environmental impact.

### 11.3 Cosmetics, textiles, and bioplastics

The cosmetic industry increasingly utilizes microbial pigments as natural alternatives to synthetic dyes due to their safety, biodegradability, and additional functional benefits. These pigments are used in skincare products, lipsticks, creams, and sunscreens because of their antioxidant and photoprotective properties, which help protect skin from oxidative stress and UV damage (Kiki, 2023). In the textile industry, microbial pigments such as prodigiosin and carotenoids are used as eco-friendly dyes for coloring fabrics, including cotton and silk. These pigments not only provide stable coloration but also impart antimicrobial properties, enabling the development of protective textiles and medical fabrics. Additionally, microbial pigments are increasingly being incorporated into biodegradable plastics and polymer materials, enhancing esthetic properties while supporting sustainable material development. Their compatibility with biodegradable matrices makes them ideal for eco-friendly packaging applications (Lopes and Ligabue-Braun, 2021; Yaashikaa et al., 2022; Devi et al., 2025a; Singhania et al., 2009).

### 11.4 Emerging applications and multifunctional pigments

Beyond conventional applications, waste-derived microbial pigments are gaining importance in emerging technological and industrial fields. These pigments are being explored as biosensors, fluorescent markers, and diagnostic agents due to their optical and electrochemical properties. Their redox activity and biofunctional properties also make them suitable for applications in nanotechnology, bioelectronics, and environmental remediation (Grewal et al., 2022). Furthermore, multifunctional pigments such as melanin and carotenoids have shown potential in bioplastics, smart packaging, and biomedical devices due to their UV-protective, antioxidant, and conductive properties. Waste-based microbial pigment production enhances sustainability while enabling the development of multifunctional biomaterials for advanced industrial applications (Lopes and Ligabue-Braun, 2021).

## 12 Techno-economic and life cycle assessment

Techno-economic analysis (TEA) and life cycle assessment (LCA) are indispensable frameworks for evaluating the commercial feasibility, environmental performance, and long-term sustainability of microbial pigment production systems, particularly those based on waste valorization. While advances in fermentation strategies, strain engineering, and bioprocess optimization determine biological productivity, TEA and LCA provide systems-level insight into whether these processes can compete with conventional synthetic and plant-derived pigments in real-world industrial and environmental contexts (David et al., 2025). Together, these complementary tools enable holistic assessment of cost drivers, environmental trade-offs, and sustainability hotspots across the pigment value chain.

### 12.1 Cost–benefit analysis of waste-based pigment production

Techno-economic analysis (TEA) evaluates the economic performance of a bioprocess by quantifying capital expenditure (CAPEX), operational expenditure (OPEX), revenue streams, and market sensitivity. In microbial pigment production, major cost contributors include feedstock procurement, fermentation operations, downstream processing, energy consumption, and effluent treatment (David et al., 2025). The use of waste-derived substrates, such as agro-industrial residues and food-processing effluents, substantially reduces feedstock costs—often the largest fraction of OPEX in conventional fermentation systems relying on refined sugars and nitrogen sources. TEA studies on waste-based bioprocesses, including carotenoid production from fruit and vegetable wastes, have projected up to 50–60% reductions in production costs when processes are optimized at industrial scale (Rasool, 2025). However, these economic gains are often partially offset by higher initial CAPEX associated with specialized extraction, purification, and solvent recovery infrastructure. Importantly, TEA models typically incorporate minimum selling price (MSP), break-even timelines, and sensitivity analyses to fluctuations in feedstock availability, energy prices, and product yield. Such analyses demonstrate that microbial pigment production can become economically competitive with synthetic alternatives under scenarios involving stable waste supply chains, efficient downstream processing, and scale-up integration (David et al., 2025). Beyond direct costs and revenues, cost–benefit analysis also considers external economic benefits, including reduced landfill disposal costs, avoided environmental remediation expenses, and the generation of co-products such as residual biomass for bioenergy or biopolymer production. These indirect benefits are particularly significant in circular bioeconomy frameworks, where waste streams are transformed from liabilities into value-generating resources.

### 12.2 Life cycle assessment and environmental impact

Life cycle assessment (LCA) quantifies the environmental impacts of a product or process across its life cycle, typically from cradle-to-gate or cradle-to-grave, using indicators such as global warming potential (GWP), cumulative energy demand, eutrophication potential, acidification, and resource depletion. LCA is especially valuable for waste-based microbial pigment production, as it captures the net environmental consequences of substituting virgin raw materials with organic waste streams (Bałdowska-Witos et al., 2023). Several LCA studies on microalgae and microbial systems integrated with wastewater treatment have demonstrated substantial environmental benefits. For example, coupling pigment recovery with wastewater remediation has been shown to reduce overall environmental impacts by up to five-fold compared with conventional cultivation on synthetic media, largely due to nutrient recycling, reduced freshwater use, and avoidance of chemical fertilizers. These systems deliver not only pigments but also critical ecosystem services, including nutrient removal and pollution mitigation (Bałdowska-Witos et al., 2023). Nevertheless, LCA outcomes are often context-dependent. Comparative LCAs of natural astaxanthin produced by microalgae and its synthetic counterpart have revealed that synthetic pigments may exhibit lower impacts in certain categories, such as energy consumption, owing to highly optimized industrial chemical processes. However, these results are highly sensitive to system boundaries, electricity mix, cultivation parameters, and assumptions regarding waste utilization and renewable energy integration (Bałdowska-Witos et al., 2023). Key environmental indicators commonly assessed in pigment LCAs include global warming potential, photochemical ozone formation, acidification potential, and abiotic resource depletion, which together reflect impacts on climate change, air quality, and long-term resource sustainability. Sensitivity and scenario analysessuch as comparing fossil-based versus renewable electricity are therefore essential for generating robust and policy-relevant conclusions.

### 12.3 Comparison with synthetic and plant-derived pigments

Comparative sustainability assessments are critical for determining whether microbial pigments offer tangible advantages over synthetic and plant-derived colorants. Synthetic pigments, largely produced from petrochemical feedstocks, benefit from high yields, consistency, and mature industrial infrastructure. However, their production is often associated with greenhouse gas emissions, toxic intermediates, and persistent environmental pollutants, particularly across upstream chemical synthesis and downstream disposal phases (Chavan et al., 2025). Plant-derived natural pigments, while generally perceived as safer and more biodegradable, face sustainability challenges related to land use, high water demand, seasonal variability, and low pigment yields, which can undermine their environmental performance at scale. These limitations are particularly pronounced when pigments compete with food crops or require extensive agricultural inputs (Gupta et al., 2026). Life cycle comparisons such as those conducted for astaxanthin indicate that although synthetic pigments may outperform natural alternatives in narrowly defined impact categories, the broader sustainability context, including non-renewable resource dependence, human health risks, and long-term ecological effects, often favors biologically derived pigments. The environmental advantage of microbial pigments becomes especially pronounced when waste streams, renewable energy, and integrated biorefineries are employed, as demonstrated by wastewater-based LCA case studies showing significantly reduced overall impacts compared with conventional systems.

In summary, although microbial pigment production faces challenges related to energy intensity, downstream processing costs, and feedstock variability, techno-economic and life cycle assessments consistently demonstrate that circular, waste-integrated approaches can deliver compelling economic and environmental advantages over traditional pigment production routes. These analyses not only guide research and development priorities but also provide critical evidence for industry stakeholders and policymakers seeking sustainable and resilient colorant solutions.

### 12.4 Commercialization bottlenecks and industrial success cases

#### 12.4.1 Yield-to-cost ratio and economic competitiveness

Despite growing industrial interest, microbial pigments remain substantially more expensive than synthetic dyes due to lower volumetric productivity, expensive downstream processing, and stringent purity requirements (Devi et al., 2024). Synthetic colorants are typically produced at large scale through highly optimized petrochemical routes with low manufacturing costs, whereas microbial pigment production involves biologically constrained fermentation processes, longer production cycles, and energy-intensive extraction (Negi, 2025). Downstream processing alone may account for approximately 60–70% of total production costs. However, integration of low-cost waste substrates, metabolic engineering, continuous fermentation systems, and biorefinery approaches may significantly improve yield-to-cost ratios and economic feasibility (Rațu et al., 2023; Możejko-Ciesielska, 2025).

#### 12.4.2 Bioreactor scale-up constraints

The transition from laboratory-scale fermentation to industrial-scale production presents substantial engineering challenges. High-cell-density fermentation frequently encounters oxygen transfer limitations due to insufficient volumetric oxygen transfer coefficients (kLa), particularly for aerobic pigment-producing microorganisms. Strategies including increased aeration, oxygen-enriched air, oxygen vectors, optimized impeller configurations, and airlift bioreactors may improve oxygen availability (Devi et al., 2024). Additionally, excessive agitation can induce shear stress, damaging shear-sensitive fungi, yeasts, and filamentous microorganisms, thereby reducing pigment productivity. Low-shear impellers, rheology optimization, immobilized cell systems, and fed-batch cultivation may help mitigate these effects (Pal et al., 2024).

Strict contamination control is another major challenge in large-scale bioprocesses, especially when utilizing heterogeneous waste-derived feedstocks that may harbor competing microorganisms (Reddy et al., 2023). Closed bioreactor systems, sterilization protocols, adaptive microbial strains, biosensors, and real-time monitoring systems are increasingly employed to maintain process integrity and reproducibility (Rațu et al., 2023).

#### 12.4.3 Pigment stability and physicochemical limitations

Pigment stability critically determines shelf life, processability, and industrial applicability. Carotenoids are highly susceptible to oxidative degradation, heat, and light exposure due to their polyunsaturated structure, whereas phycobiliproteins are particularly sensitive to pH and temperature fluctuations (Lad et al., 2022). Melanins exhibit comparatively high thermal and oxidative stability but may show variable solubility depending on polymer composition. Prodigiosin and violacein are prone to photodegradation and solvent instability under harsh processing conditions (Możejko-Ciesielska, 2025). Stabilization strategies such as microencapsulation, nanoemulsions, co-pigmentation, antioxidant addition, and modified atmosphere packaging have shown promising results in improving pigment durability and preserving bioactivity (Panesar et al., 2015; Usmani et al., 2020).

#### 12.4.4 Industrial success cases

Several microbial pigments have demonstrated commercial feasibility. *β*-Carotene produced by *Blakeslea trispora* has achieved industrial success as a food colorant and nutraceutical ingredient (Rather et al., 2023). Phycocyanin derived from *Arthrospira platensis* is commercially marketed as a natural blue food colorant and fluorescent biomolecule (Galasso et al., 2017). Monascus pigments produced through agro-industrial fermentation have long been utilized in fermented food products in Asia, while microbial astaxanthin derived from *Xanthophyllomyces dendrorhous* and microalgal systems has gained substantial value in aquaculture, cosmetics, and nutraceutical sectors (Tran-Ly et al., 2020). These examples demonstrate that although commercialization barriers persist, targeted process optimization and circular bioprocess integration can facilitate industrial adoption of microbial pigments (Pandey et al., 2023).

### 12.5 Digitalization, artificial intelligence, and process control in circular bioprocessing

The integration of digitalization and artificial intelligence (AI) into biotechnological workflowsoften referred to as *Bioprocessing 4.0*is emerging as a transformative enabler for sustainable microbial pigment production. Conventional bioprocess development relies heavily on empirical optimization and sequential experimentation, which is both time-consuming and resource-intensive, particularly when dealing with heterogeneous waste-derived substrates. In contrast, digital tools such as machine learning (ML), digital twins, advanced sensors, and adaptive control systems enable data-driven, predictive, and autonomous process optimization (Możejko-Ciesielska, 2025).

AI driven models can analyze large, multidimensional datasets generated during fermentation to identify key performance determinants, including substrate uptake kinetics, oxygen transfer demand, metabolic flux distribution, and pigment biosynthesis rates. Supervised and unsupervised ML algorithms have been successfully applied to predict optimal fermentation conditions, significantly reducing experimental workloads while improving reproducibility and robustness across batches (Helleckes et al., 2023). Such approaches are particularly valuable in circular bioprocessing, where feedstock composition and microbial responses are inherently variable. Digital twins, which are virtual replicas of physical bioreactors, represent a powerful tool for simulating process behavior under dynamic operating conditions. By integrating mechanistic models with real-time sensor data, digital twins can forecast system responses to changes in feedstock composition, aeration, or temperature, thereby improving process control and scale-up reliability (Helleckes et al., 2023). These virtual environments also allow in silico testing of operational strategies that would be costly or risky to explore experimentally. Recent advances in reinforcement learning (RL) further extend digital control capabilities by enabling bioreactors to autonomously adjust operating parameters such as pH, dissolved oxygen, and feed rates in response to real-time performance metrics. RL-based controllers have demonstrated resilience against process disturbances and drift, making them well suited for long-duration fermentations using waste substrates with fluctuating quality (Zhang et al., 2025). Collectively, the adoption of digitalization and AI is expected to support continuous real-time optimization, accelerated strain–process matching, and intelligent scale-up, ultimately improving productivity while reducing energy use, raw material consumption, and operational costs. Future research should prioritize the development of interoperable digital platforms that integrate upstream cultivation data, metabolic performance indicators, and downstream recovery metrics into unified decision-support systems.

### 12.6 Roadmap toward sustainable commercialization

The successful commercialization of microbial pigments requires a coordinated, systems-level roadmap that addresses scientific, technological, regulatory, and societal dimensions simultaneously. While individual advances in strain engineering or fermentation optimization are essential, isolated progress is insufficient to overcome the structural barriers facing waste-based pigment bioprocesses. Instead, integrated strategies are needed to ensure scalability, regulatory compliance, and market acceptance (Sundararajan and Ramasamy, 2024). Standardization of waste feedstocks and preprocessing protocols represents a critical first step. Harmonized methods for waste characterization, pretreatment, and conditioning can substantially reduce variability in fermentation performance across seasons and geographical locations. Such standardization is essential for reproducibility, regulatory approval, and industrial reliability (Ungureanu et al., 2025). Advanced strain engineering remains a central driver of competitiveness. The development of robust microbial cell factories with enhanced pigment yield, broad substrate utilization capacity, and tolerance to inhibitory compounds will be essential for efficient waste valorization. The convergence of CRISPR-based genome editing, systems biology, and AI-guided design is expected to accelerate strain optimization by enabling predictive identification of metabolic bottlenecks and rational pathway redesign (Molla and Meseret, 2025). Embedding techno-economic analysis (TEA) and life cycle assessment (LCA) early in process development is equally critical. These tools provide quantitative guidance on cost drivers, environmental trade-offs, and scalability constraints, enabling informed decision-making during strain selection, reactor design, and downstream processing. Early integration of TEA and LCA can prevent costly redesigns and align research trajectories with sustainability and investment priorities (Molla and Meseret, 2025).

Regulatory alignment and market readiness constitute another major pillar of the commercialization roadmap. Close collaboration among researchers, industry stakeholders, and regulatory agencies is required to establish clear safety, quality, and labeling standards for microbial pigments, particularly those derived from engineered strains or waste substrates. Harmonized regulatory frameworks can reduce approval timelines and foster international market access.

Finally, stakeholder engagement and consumer education are indispensable for long-term adoption. Building trust among feedstock suppliers, manufacturers, and consumers requires transparent communication of the benefits of microbial pigments, including non-toxicity, biodegradability, reduced environmental footprint, and multifunctional properties such as antioxidant or antimicrobial activity. Positioning microbial pigments not merely as colorants but as *functional bio-based ingredients* can differentiate them from synthetic alternatives and strengthen their value proposition.

## 13 Conclusion

Microbial pigments are emerging as sustainable alternatives to synthetic dyes for applications in food, cosmetics, pharmaceuticals, textiles, and biomedicine. This review demonstrates that integrating microbial biotechnology with waste valorization and circular bioeconomy principles enables environmentally responsible and economically viable pigment production.

The metabolic diversity of pigment-producing microorganisms supports the biosynthesis of carotenoids, melanins, prodigiosin, violacein, and phycobiliproteins using agro-industrial, food, lignocellulosic, marine, and municipal waste streams as low-cost substrates. Such waste-to-wealth strategies reduce reliance on refined media while addressing critical waste management challenges.

Advances in pretreatment, fermentation, metabolic engineering, and green downstream processing have improved yield, robustness, and sustainability. Techno-economic and life cycle assessments further indicate that optimized waste-based processes can outperform conventional synthetic and plant-derived pigments in environmental impact and cost.

Remaining challenges feedstock variability, scalability, regulatory complexity, and consumer perception require coordinated solutions involving process standardization, digitalization, AI-assisted control, and regulatory alignment. Overall, microbial pigments produced through circular bioprocesses represent a promising pathway toward sustainable industrial colorant production.
